# Distinct cellular and reproductive consequences of meiotic chromosome synapsis defects in *syce2* and *sycp1* mutant zebrafish

**DOI:** 10.1371/journal.pgen.1011656

**Published:** 2025-09-05

**Authors:** Iván Olaya, Ilara N. Yilmaz, Naima Nour-Kasally, Ross E. Charboneau, Bruce W. Draper, Sean M. Burgess

**Affiliations:** 1 Department of Molecular and Cellular Biology, University of California Davis, Davis, California, United States of America; 2 Integrative Genetics and Genomics Graduate Group, University of California Davis, Davis, California, United States of America; National Cancer Institute, UNITED STATES OF AMERICA

## Abstract

The synaptonemal complex (SC) is a meiosis-specific structure that aligns homologous chromosomes and promotes the repair of meiotic DNA double-strand breaks (DSBs). To investigate how defects in SC formation affect gametogenesis in zebrafish, we analyzed mutations in two genes encoding core SC components: *syce2* and *sycp1*. In *syce2* mutants, chromosomes exhibit partial synapsis, primarily at sub-telomeric regions, whereas *sycp1* mutant chromosomes display early prophase co-alignment but fail to synapse. Both mutants exhibit reduced efficiency in repairing meiotic DSBs compared to wild type. Despite these defects, *syce2* and *sycp1* mutant females are fertile. However, *sycp1* mutant females produce a higher proportion of malformed progeny, correlating with increased univalent formation. While *syce2* mutant males are fertile and produce normal offspring, *sycp1* mutant males are sterile, with spermatocytes that transit prophase I but arrest at metaphase I or II. Additionally, *sycp1* mutants display a male-biased sex ratio that can be suppressed by extending the developmental window for sex determination, suggesting that the absence of synapsis delays-but does not completely block-meiotic progression. Notably, embryos from *syce2* and *sycp1* mutant females exhibit widespread somatic mosaic aneuploidy, indicating that impaired meiotic chromosome dynamics can compromise genome stability during early development. In contrast to mouse SC mutants, the zebrafish *syce2* and *sycp1* mutants examined in this study progress through meiotic prophase I with minimal disruption, suggesting a less stringent surveillance mechanism for synapsis errors in zebrafish.

## Author summary

Meiosis is a specialized cell division that generates eggs and sperm and ensures that offspring inherit the correct number of chromosomes. A protein-based structure called the synaptonemal complex (SC) plays a central role in aligning homologous chromosomes and promoting the repair of intentional DNA breaks that occur during meiosis. Defects in the SC can lead to infertility or birth defects, but the consequences of these defects can vary across species. In this study, we used Zebrafish to investigate how mutations in two core SC genes, *syce2* and *sycp1*, affect meiosis and development. We found that both genes are required for proper chromosome synapsis and DNA repair during meiosis, but the consequences of disrupting each gene differ. Mutants lacking *sycp1* are sterile as males and produce malformed embryos as females, while *syce2* mutants are fertile and produce healthy offspring. Surprisingly, despite their meiotic defects, both mutants proceed through meiosis without triggering a strong arrest, suggesting that zebrafish may have a weaker surveillance system than mammals. Furthermore, we found that offspring from mutant females often show mosaic chromosome abnormalities, indicating that meiotic errors can lead to genome instability during early development. These findings highlight important differences in how vertebrates monitor meiosis and reveal a previously underappreciated link between meiotic chromosome regulation and somatic genome stability.

## Introduction

There has been a world-wide trend of decreasing sperm count among men, yet the cause is unknown [1]. Mutations in genes encoding protein components of the meiotic synaptonemal complex (SC) are associated with nonobstructive azoospermia [2–5] in men and pregnancy loss [6] and primary ovarian insufficiency syndrome in women [7,8]. It is possible that effects of less deleterious mutations might be enhanced by environmental factors [1]. Studies in several model organisms have demonstrated the critical role the SC plays in the completion of meiosis that have provided insights into human infertility [9].

Meiosis is a specialized cellular program that is required to produce haploid gametes through one round of DNA replication and two consecutive rounds of chromosome segregation. Homologous chromosomes (homologs) are segregated in meiosis I while sister chromatids are segregated in meiosis II. Crossing over between non-sister chromatids of homologs by homologous recombination is central to proper meiosis I segregation. Recombination is initiated by forming and repairing programmed DNA double-strand breaks (DSBs) that are catalyzed by the enzyme Spo11 [10,11]. Each homolog pair experiences at least one crossover that supports proper segregation [12–17]. This so-called “crossover assurance” depends on ZMM (Zip, Msh, Mer) proteins that function within the context of the synaptonemal complex (SC) [18,19].

**Fig 1:**
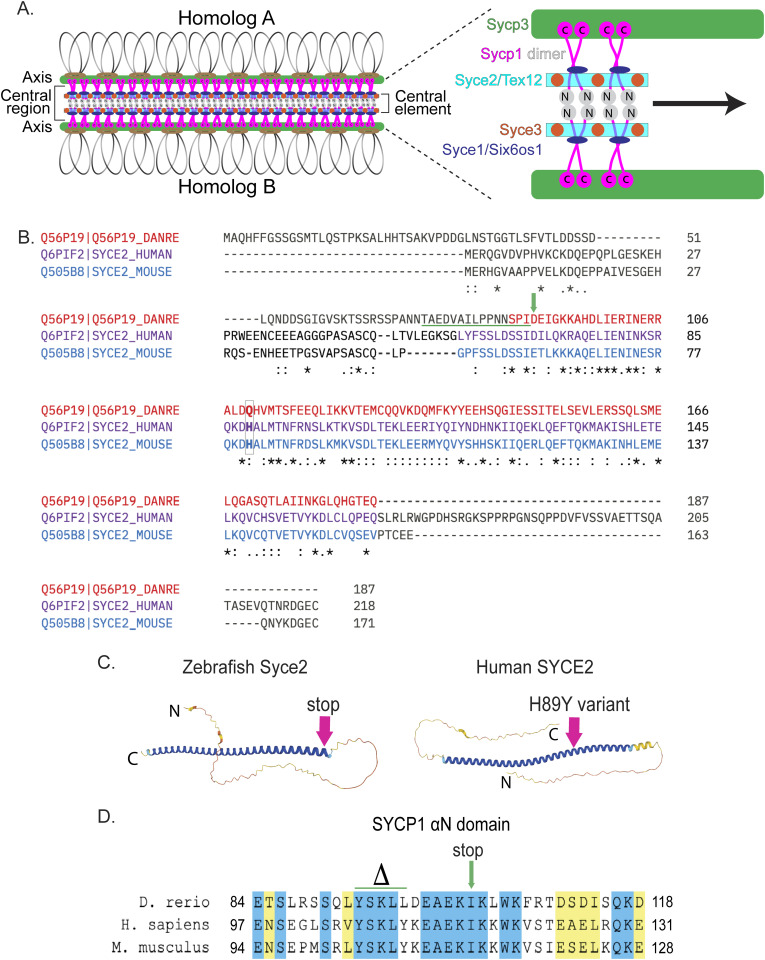
Generating the *syce2* and *sycp1* mutants. (A) Schematic of the synaptonemal complex showing key structures of meiotic chromosomes: DNA loops (gray/black), chromosome axis (Sycp3, green), central region (Sycp1, magenta) and the central element components (Syce2/Tex12, cyan; Syce1/Six6os1, dark blue; and Syce3, orange) that includes the Sycp1 N-termini (gray). (B) Alignment of the zebrafish Syce2 amino acid sequence with human and mouse orthologs using the Clustal Omega multiple sequence alignment tool [124]. Colored text defines regions that form a long *α* helix in a 2:2 complex [26]. Conserved identical residues (*), strongly similar (:) and weakly similar (.). The amino acids (aa) affected by the frameshift mutation are underlined in green. The position of the premature stop codon in *syce2*^*-/-*^ is shown by a green arrow. The relative position of the human mutation (H89Y) associated with pregnancy loss [6] is highlighted in the boxed region. (C) AlphaFold-predicted structures for Syce2 protein from zebrafish (AF-Q56P19-F1) and human (AF-Q6PIF2-F1) [128]. The position of the 89th codon for zebrafish Syce2 that generates the premature stop codon is shown by a pink arrow. The position of the human variant in SYCE2 is depicted by the pink arrow. (D) Alignment of aa comprising the *α*N domain of Human SYCP1 that promotes self-assembly [25]. Blue regions are identical aa and yellow regions are similar aa. The CRISPR generated *sycp1* mutation is a complex mutation with a deletion that removes the amino acids marked by delta and an insertion that truncates the protein at the amino acid marked by the arrow. See S1 Fig for details.

The SC is an evolutionarily conserved tripartite structure observed in meiotic prophase across many species [20] and is composed of two chromosome axes, one for each homolog, a central region containing proteins that connect homologous axes, and a central element within the central region that stabilizes the overall structure (Fig 1A) [21,22]. In vertebrates, the central region contains head-to-head interacting dimers of the transverse filament protein SYCP1 flanked by two bands of central element filaments containing SYCE1, SYCE2, SYCE3, TEX12 and SIX6OS1 [21]. Zebrafish share orthologs of corresponding genes with mouse and humans. In mouse, SYCE2 and TEX12 form a complex that stabilizes interactions between the alpha-helical domains at the N terminus (𝛼N) of SYCP1 [23–27]. Once the SC is established at sites of future crossovers, the central element promotes longitudinal expansion of the SC to align chromosomes end-to-end. Mouse SYCE2 is required to fully assemble the SC by stabilizing SYCP1 interactions, and its absence leads to foci or short stretches of SYCP1 between paired chromosome axes [23]. Functional orthologs of the central element proteins also exist in the SC in budding yeast [28] and *Drosophila* [29], highlighting the conserved nature of SC assembly.

A meiotic checkpoint network exists to coordinate the ordered execution of dependent events of meiotic prophase I [30]. Asynapsis or unrepaired DSBs can lead to delayed or arrested prophase I, and in some cases result in apoptosis. In mice, the failure to fully synapse homologs activates a checkpoint that is separable from one surveilling DSBs, and in both cases cells are eliminated at meiotic prophase I [31–35]. In humans, mutations in the central element gene *SYCE1* are associated with nonobstructive azoospermia [2,4]. Synapsis defects in other model organisms tend to have less severe phenotypes: In *C. elegans*, a null mutation in the *sycp1* ortholog, *syp-1*, results in an increase in apoptosis in the germline [36,37] and egg production is reduced by ~22–24% [38,39]. In budding yeast, disrupting the transverse filament or central element of the SC delays the first meiotic division, presumably due to the slow repair of DSBs and not through a synapsis checkpoint *per se* [40–44]. In *Drosophila* females, partial disruption of the SC leads to a checkpoint-mediated prophase I delay, yet total loss of SC does not appear to cause delay or arrest [45,46]. In *Arabidopsis*, elimination of the SC has marginal effects on seed number and pollen viability [47]. Zebrafish *spo11* mutant females produce normal numbers of fertile eggs despite defects in DSB formation and synapsis [48]. However, it is possible that a “checkpoint-inducing” signal is not produced in the *spo11* mutant, similar to what has been proposed for the *spo11* mutant in budding yeast [49,50]. In this study, we investigated how loss of synapsis in *syce2* and *sycp1* mutant zebrafish impacts meiotic progression and reproduction and compare and contrast outcomes with these model organisms.

The chromosome events of meiotic prophase I follow a coherent timeline in zebrafish [48,51]. During leptotene, the deposition of short axial structures at each telomere is concurrent with the formation of DSBs. At early zygotene, co-alignment of short homolog axes is followed by the initiation of the SC, which extends closely behind the growing axes creating a moving fork until homologs are synapsed from end-to-end at pachytene. Co-alignment and synapsis appear to initiate almost exclusively at the ends of chromosomes while interstitial regions are joined by “zippering up”. This is reminiscent of the temporal order of events in *C. elegans*, where pairing and synapsis are initiated solely at defined pairing centers located at one end of each chromosome and the SC extends in a linear fashion [52–54]. The important difference is that in *C. elegans*, pairing at chromosome ends is mediated by protein/protein interactions to bridge homologs, while in zebrafish it is mediated by the processing of Spo11-induced DSBs.

A mutation in the zebrafish *sycp1* gene was recovered in a forward genetic screen to identify mutants defective for spermatogenesis [55]. Further analysis of mutant *sycp1*^*isa*/isa^ males showed that homologs pair at the ends but do not synapse [56]. In addition, spermatogenesis was arrested, though the meiotic stage of the arrest was not determined. Since females were not analyzed in this study, it remained unknown how loss of *sycp1* affects oogenesis. Here, we examined zebrafish containing loss-of-function mutations in *sycp1* or *syce2* and found that synapsis is completely abolished in *sycp1*^*-/-*^ mutants and reduced to short synapsed-like structures at the ends of chromosomes in *syce2*^*-/-*^ mutants. Both mutants exhibit increased numbers of DSBs in zygotene nuclei and inefficient repair implicating the SC in this process. Remarkably, *syce2*^*-/-*^ mutant males and females are fertile and produce a high percentage of healthy offspring. By contrast, *sycp1*^*-/-*^ males are infertile with spermatocytes transiting prophase I to metaphase. In addition, *sycp1*^*-/-*^ females produce normal numbers of eggs but predominantly malformed progeny, though some live to adulthood. Our study highlights the differences in reproductive outcomes between males and females in a teleost species in response to synapsis defects. We also show that embryos of *syce2*^*-/-*^ and *sycp1*^*-/-*^ mothers exhibit a striking degree of somatic mosaic aneuploidy suggesting that defects in meiotic genes can lead to genome instability during embryogenesis.

## Results

### Targeted mutation of *syce2* and *sycp1*

The zebrafish *syce2* gene consists of 5 exons that encode a 187 amino acid (aa) protein. Alignment of the zebrafish Syce2 amino acid sequence with human and mouse orthologs highlights conservation of the core region of SYCE2 in vertebrates (Fig 1B). The core is a long α helical structure shown to interact with TEX12 [24]. A human *SYCE2* genetic variant associated with pregnancy loss is located within the core region [6] (Fig 1B). To disrupt the function of zebrafish *syce2*, we used CRISPR-Cas9 to introduce an indel mutation resulting in a predicted 88 aa truncated protein (*syce2*^*uc98*^; Figs 1B-green arrow; and S1A). An AlphaFold-predicted structure shows that the *syce2*^*uc98*^ mutation should remove all but three amino acids of the core and abolish the Tex12 interaction domain (Fig 1C). Antibodies to mammalian SYCE2 did not cross-react with the zebrafish protein. In this study we refer to the *syce2*^*uc98/uc98*^ mutant as *syce2*^-/-^.

The *sycp1* gene contains 32 exons that encode a 1000 aa protein. Using CRISPR-Cas9 we introduced a deletion/insertion mutation into exon 5 (*sycp1*^*uc97*^; S1B Fig). This mutation creates a net five aa deletion within the αN domain immediately followed by a premature stop codon resulting in a predicted 97 aa protein (Fig 1D). For this study we refer to the *sycp1*^*uc97/uc97*^ mutant as *sycp1*^*-/-*^. Sequencing of RT-PCR products generated from *syce2*^*-/-*^ and *sycp1*^*-/-*^ testes RNA confirmed that the mutations do not cause exon skipping that could potentially lead to a truncated but functional protein (S1C Fig) [57,58]. Immunostaining Sycp1 reveals its localization is absent in *sycp1*^*-/-*^ mutants (described below).

### Loss of Syce2 leads to asynapsis of interstitial regions of homologous chromosomes

**Fig 2:**
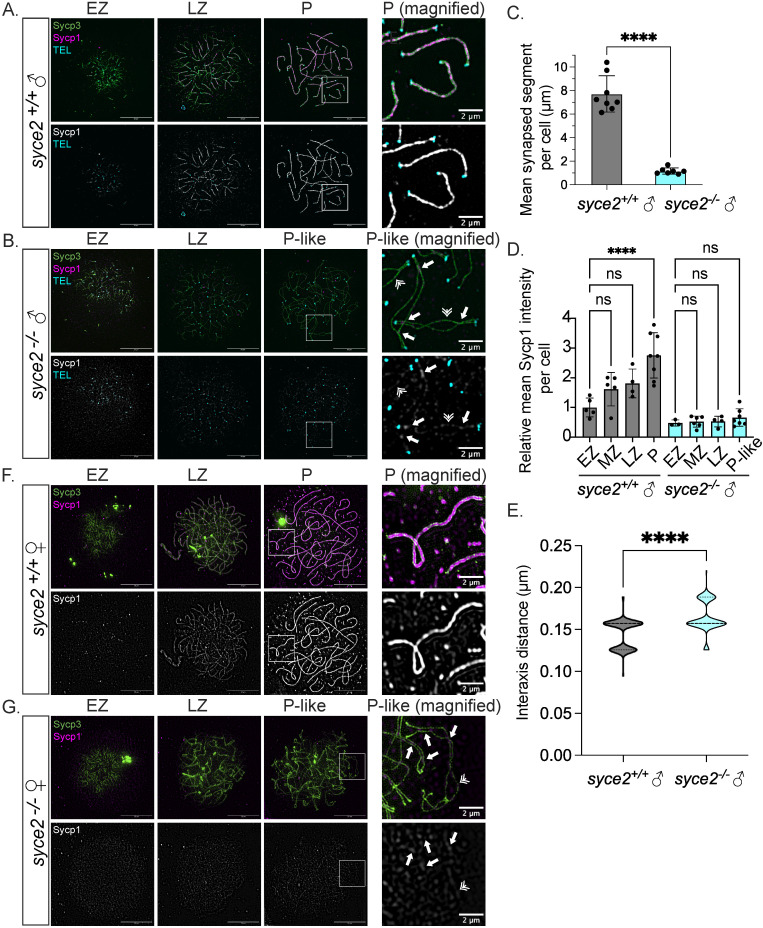
Loss of Syce2 function results in asynapsed interstitial regions. (A–B) Surface-spread chromosomes from *syce2*^+/+^ (A) and *syce2*^*-/-*^ (B) spermatocytes imaged using structured illumination microscopy. Proteins are immunostained for Sycp3 (green) and Sycp1 (magenta and gray). Telomeres (cyan) are detected by *in-situ* hybridization using a fluorescent PNA probe. Examples of spread chromosomes during meiotic prophase I are shown: Early Zygotene (EZ), Late Zygotene (LZ), Pachytene (P) (*syce2*^+/+^), and Pachytene-like (P-like) (*syce2*^*-/-*^). Scale bar = 10 µm. Boxed region represents magnified examples of synapsed pachytene (*syce2*^+/+^) and pachytene-like (*syce2*^*-/-*^) chromosomes. Scale bar for magnified examples = 2 µm. Arrows represent synapsed-like regions with Sycp1 localization. Double arrowheads indicate Sycp1 localization at unaligned axial regions. (C) Mean synapsed segment per cell (µm) in pachytene and pachytene-like spermatocytes. The mean synapsed segment is significantly lower in *syce2*^-/-^ spermatocytes (n = 7) compared to *syce2*^+/+^ (n = 8). Significance was determined using an unpaired t-test. **** = p < 0.0001. (D) Relative mean Sycp1 intensity per cell from early zygotene to pachytene or pachytene-like spermatocytes normalized to the mean *syce2*^+/+^ EZ values. Mean-fluorescence intensity between the axes (n = 25) – mean background (n = 6) was determined for each cell using the segmented-line tool in ImageJ. n = 5, *syce2*^+/+^ – EZ; n = 5; *syce2*^+/+^ – MZ; n = 4, *syce2*^+/+^ – LZ; n = 8, *syce2*^+/+^ – P; n = 3, *syce2*^-/-^ – EZ; n = 6, *syce2*^-/-^ – MZ; n = 4, *syce2*^-/-^ – LZ; n = 7, *syce2*^-/-^ – P-like. Significance was determined using ordinary one-way ANOVA testing with Šidák’s multiple comparisons test. ns = not significant; **** = p < 0.0001. (E) Distance between axes for co-aligned axial pairs in pachytene (n = 126) and pachytene-like (n = 93) spermatocytes from 8 cells in *syce2*^+/+^ and 7 cells in *syce2*^*-/-*^ shown as a violin plot. Note that one pixel is equal to 0.03 µm resulting in a graph with a bimodal distribution. Significance was determined using an unpaired t-test. **** = p < 0.0001. (F–G) Surface-spread chromosomes from *syce2*^+/+^ (F) and *syce2*^*-/-*^ (G) oocytes imaged using structured illumination microscopy immunostained for Sycp3 and Sycp1. Examples of spread chromosomes during meiotic prophase I are described in (A–B). Scale bar = 10 µm. Boxed region represents magnified examples of synapsed pachytene and pachytene-like chromosomes. Scale bar for magnified examples = 2 µm. Arrows represent synapsed-like regions with Sycp1 localization. Double arrowhead indicates Sycp1 localization at unaligned axial regions.

Since mammalian SYCE2 functions to extend the SC from sites of initiation [23], we predicted that in *syce2*^*-/-*^ zebrafish, synapsis would occur at the ends of chromosomes but would fail to “zipper-up” end-to-end. To test this, we analyzed images of immunostained surface-spread chromosomes from wild-type and *syce2*^*-/-*^ zebrafish spermatocytes with antibodies to Sycp3, Sycp1, and a probe hybridizing to telomeres, and imaged them using structured-illumination microscopy. In wild-type spermatocytes, the homolog axes assemble initially at the chromosome ends in leptotene, co-align in early zygotene, then extend concurrently with the deposition of Sycp1 to form full-length SC in pachytene (Figs 2A and S2A). In *syce2*^*-/-*^ spermatocytes, the majority of the 50 chromosome ends form short parallel axial tracks in early zygotene, suggesting that homologs have found their partners (Figs 2B and S2B). However, the synapsed-like configuration at the ends does not lengthen as meiotic prophase I progresses. Instead, the axes continue to elongate until they include the entire length of each homolog, but the interstitial regions remain separated. Occasional patches of synapsed-like axes occur in the interstitial regions in the *syce2*^*-/-*^ mutant but are relatively rare. We measured the mean lengths of synapsed and synapsed-like segments per nucleus which are 7.71 ± 1.54 µm in wild-type pachytene nuclei compared to 1.16 ± 0.26 µm in pachytene-like nuclei of the mutant (Fig 2C). These results indicate that short stretches of SC form between homologs in the absence of Syce2 but that longitudinal extension of the SC is inhibited.

To better understand how synapsed-like regions compare to *bona fide* SC, we measured the relative levels of Sycp1 protein in synapsed (wild-type) and synapsed-like (*syce2*^*-/-*^) chromosomes in nuclei that represented all stages of prophase I. In wild-type spermatocytes, the relative mean Sycp1 fluorescence intensity increased steadily by nearly 3-fold by the end of prophase I from 1.00 ± 0.32 in early zygotene to 2.75 ± 0.76 in pachytene (Fig 2D). In *syce2*^*-/-*^ spermatocytes, Sycp1 also loads between nascent axes, but at levels barely above background, and as foci or short patches instead of continuous stretches (Fig 2B; P-like magnified). Sycp1 is also found at low levels along the lengths of asynapsed axes by visual inspection. In addition, the relative mean intensity of Sycp1 between axes in *syce2*^*-/-*^ spermatocytes remains low from early zygotene (0.48 ± 0.10) through the pachytene-like stage (0.66 ± 0.29) (Fig 2D). The diminished Sycp1 levels observed in the *syce2*^*-/-*^ mutant presumably reflect the destabilization of the central region.

We next tested whether the reduced level of Sycp1 on *syce2*^*-/-*^ chromosomes affects the distance between axes in the synapsed-like regions. For this, a line perpendicular to the parallel axes was drawn in ImageJ and the distance between the maximum intensity signal (peaks) for each axis was determined. We found that the mean distance between peaks was slightly larger in the *syce2*^*-/-*^ mutant (0.17 ± 0.02 µm) compared to wild-type (0.14 ± 0.02 µm) (Fig 2E). These results show that the synapsed-like regions in *syce2*^*-/-*^ mutants resemble wider than normal SC with reduced amounts of Sycp1.

We next examined chromosome spreads of *syce2*^*+/+*^ and *syce2*^*-/-*^ oocytes (Fig 2F and 2G). As seen for spermatocytes, Sycp1 also appears as foci at sub-telomeric regions in *syce2*^*-/-*^ zygotene oocytes (as detected by bright nodules of Sycp3 staining) and as short stretches in pachytene-like oocytes (Fig 2G). While we did not observe full-length chromosome alignment in the *syce2*^*-/-*^ females, the co-aligned (< 0.5 µm) and synapsed-like regions are found both at the ends of chromosomes and in interstitial regions. Since the distribution of crossovers indicated by Mlh1 foci is more evenly distributed in zebrafish oocytes compared to spermatocytes [59,60], it appears that partial synapsis may mirror the different recombination landscape of the two sexes. Notably, there is considerable entanglement of chromosome axes in both oocytes and spermatocytes.

### Formation and loss of co-aligned axes in *sycp1*^*-/-*^ spermatocytes resembles wild-type chromosome behavior

**Fig 3:**
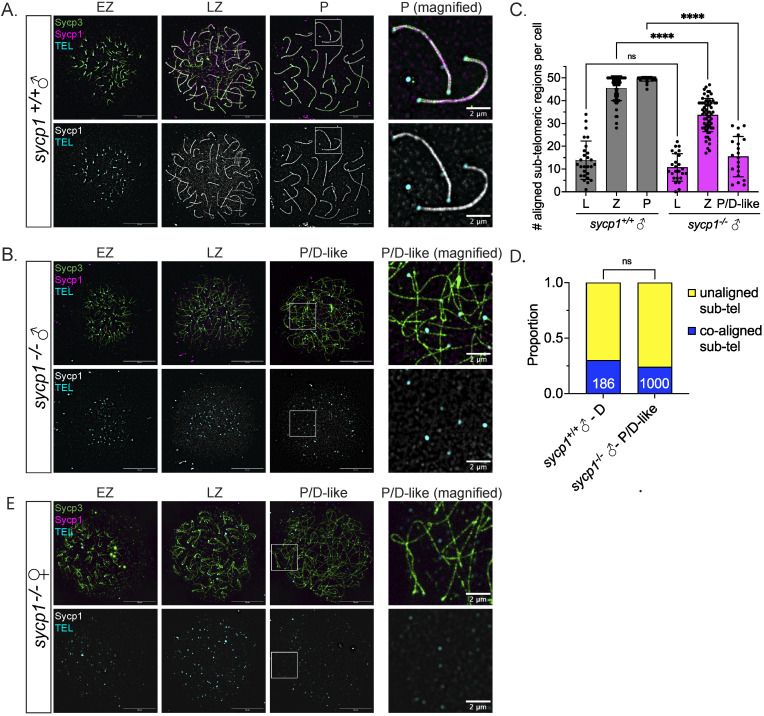
Formation and loss of co-alignment in *sycp1*^*-/-*^ spermatocytes. (A) Surface-spread chromosomes from *sycp1*^*-/-*^ spermatocytes imaged using structured illumination microscopy stained for Sycp3 (green), Sycp1 (magenta and gray) and telomeres (cyan). Examples of spread chromosomes during meiotic prophase I are shown: EZ (early zygotene), LZ (late zygotene), P (pachytene). Scale bar = 10 µm. Boxed region represents magnified examples of pachytene chromosomes. Scale bar for magnified examples = 2 µm. (B) Surface-spread chromosomes from *sycp1*^*-/-*^ spermatocytes as described in (A); P/D (pachytene/diplotene-like). Scale bar = 10 µm. Boxed region represents magnified examples of P/D-like chromosomes. Scale bar for magnified examples = 2 µm. (C) Number of co-aligned pairs of sub-telomeric regions per cell across prophase I in *sycp1*^+/+^ and *sycp1*^-/-^ spermatocytes from (A–B). Images for leptotene (L) are not shown but are characterized by predominantly short un-aligned axes. n = 27, *sycp1*^+/+^– L; n = 25, *sycp1*^-/-^– L; n, = 61 *sycp1*^+/+^– Z; n = 60, *sycp1*^-/-^– Z; n = 36, *sycp1*^+/+^– P; n = 20, *sycp1*^-/-^– P/D-like. Significance was determined using ordinary one-way ANOVA testing with Šidák’s multiple comparisons test. ns = not significant; **** = p < 0.0001. (D) Proportion of co-aligned and unaligned pairs of sub-telomeric regions in *sycp1*^+/+^ diplotene (D) (n = 186) and *sycp1*^-/-^ P/D-like (n = 1000) spermatocytes. 11 cells were used for *sycp1*^+/+^ and 20 cells for *sycp1*^-/-^. Significance was determined using Fisher’s exact test. ns = not significant. (E) Surface-spread chromosomes from *sycp1*^*-/-*^ oocytes as described in (A and B). Scale bar = 10 µm. Boxed region represents magnified examples of P/D-like chromosomes. Scale bar for magnified examples = 2 µm.

A previous study of the *sycp1*^*isa*^ allele reported that sub-telomeric regions of *sycp1*^*isa*/isa^ spermatocytes were aligned in leptotene and early zygotene, but that these tight associations were lost at late prophase stages [56]. We also found co-aligned ends during early zygotene (EZ) and late zygotene (LZ) stage of spermatogenesis that are similar to wild-type chromosome spreads (Fig 3A and 3B). We found the number of co-aligned pairs of chromosome ends per cell in *sycp1*^-/-^ leptotene spermatocytes (10.7 ± 6.1) out of 50 is comparable to wild-type (13.9 ± 8.4) (Fig 3C) and these values increase in zygotene (33.7 ± 7.3), yet they do not reach the same level as synapsed ends in wild-type (45.5 ± 5.5). Instead, the number of co-aligned regions between full-length axes, which we refer to as “pachytene/diplotene-like”, in *sycp1*^*-/-*^ mutant spermatocytes is reduced from zygotene levels by ~54% (15.5 ± 8.8) consistent with the *sycp1*^*isa*/isa^ phenotype which was attributed to possibly weak contacts at sub-telomeric regions or DSBs resolving into non-crossovers [56]. Similar to *syce2*^*-/-*^ mutants, there is considerable entanglement of axes in both oocytes and spermatocytes.

We next considered that the loss of co-aligned ends in *sycp1*^*-/-*^ pachytene/diplotene-like nuclei might reflect a natural feature of wild-type diplotene chromosomes and extended the analysis to compare pachytene/diplotene-like chromosome ends in the mutant to wild-type diplotene chromosomes. Diplotene is the stage following pachytene when the SC is disassembled, and homologs are held together by chiasmata (sites of crossing over). Thus, the proximity of telomeres in diplotene bivalents depends on the distance between a chiasma and the adjacent chromosome ends. Since zebrafish spermatocytes experience ~1.1 crossovers per chromosome on average [59,61], it would be expected that most telomeres of diplotene chromosomes will not be in close proximity. We used 0.5 µm as the maximum distance to consider chromosome axes as co-aligned as in [48] based on the ~ 400 nm co-alignment distance between axes reported in other species prior to or without synapsis [62,63]. Since meiotic chromosomes do not undergo uniform desynapsis in any given nucleus, we limited our analysis to desynapsed ends in diplotene nuclei that we define as having three or more bivalents where at least 50% of full-length axes are not synapsed (S3 Fig). The number of ends we could evaluate in the *sycp1*^*-/-*^ spermatocytes is much larger since they were all considered to be asynapsed. The proportion of desynapsed aligned ends in wild-type diplotene spermatocytes (29.6%) is indistinguishable from *sycp1*^*-/-*^ pachytene/diplotene-like spermatocytes (24.2%) (Fig 3D). Therefore, loss of sub-telomeric contacts is a general property of normal diplotene chromosomes and *sycp1*^*-/-*^ chromosomes with full-length axes may be in a diplotene-like configuration.

### Co-aligned axes but not synapsis in *sycp1*^*-/-*^ oocytes

A previous study of the *sycp1*^*isa*^ allele failed to recover females in the homozygous population [56], therefore how loss of Sycp1 affects oogenesis was not determined. Because we found females among the *sycp1*^*-/-*^ population, we could examine chromosome spreads of *sycp1*^*-/-*^ females. We found evidence of chromosome co-alignment without synapsis at sub-telomeric regions in early zygotene as in wild-type (Figs 3E and 2F), but full-length alignment is not observed in later stage oocytes. These results show that synapsis is dispensable for co-alignment but required for end-to-end alignment of homologs during oogenesis.

### Different reproductive outcomes in *syce2*^*-/-*^ and *sycp1*^*-/-*^ mutants despite severe synapsis defects

**Fig 4:**
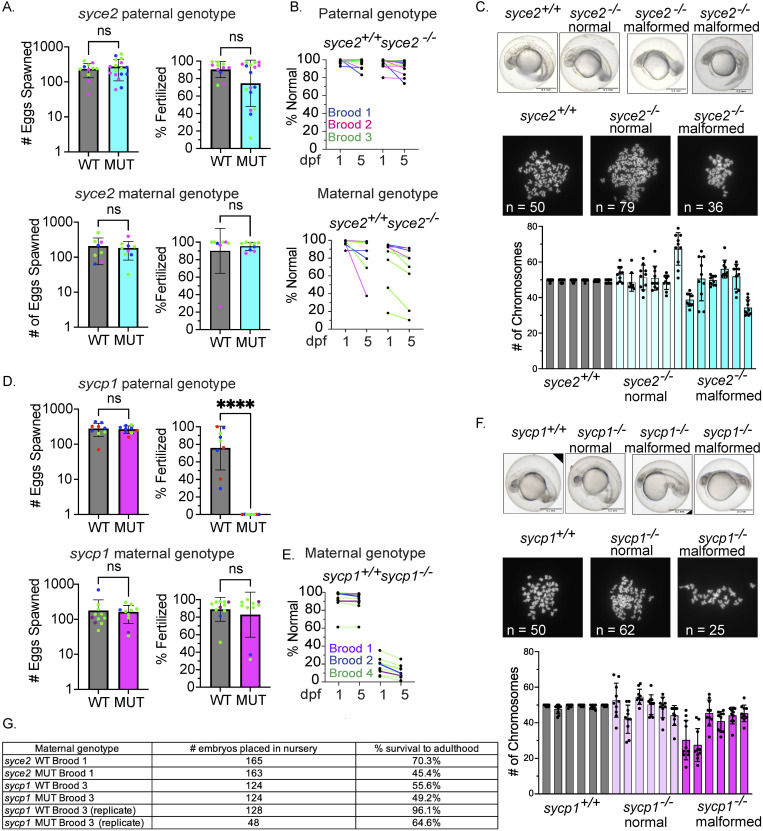
*syce2*^*-/-*^ and *sycp1*^*-/-*^ mutants have different reproductive outcomes. (A) Number of eggs spawned and fertilization rate per cross from wild-type and *syce2*^-/-^ mutants; paternal genotype (top) and maternal genotype (bottom). Each data point represents a cross yielding > 20 eggs from individual animals from three different broods born on different days. Percent fertilized excludes decomposed eggs. Each dot represents an individual animal crossed to AB wild-type fish of the opposite sex. *syce2* paternal genotype: Brood 1 (S4461; blue): WT n = 3, MUT n = 6; Brood 2 (S4562, pink): WT n = 2; MUT n = 5; Brood 3 (S4581; green): WT n = 6, MUT n = 6. *syce2* maternal genotype: Brood 1 (S4461; blue): WT n = 2, MUT n = 1; Brood 2 (S4562, pink): WT n = 2; MUT n = 2; Brood 3 (S4581; green): WT n = 4, MUT n = 5. Significance was determined using an unpaired t-test for pooled data. ns = not significant. (B) Percent Normal-looking offspring at 1- and 5-days post fertilization (dpf) from wild-type and *syce2* mutants from data in (A). Progeny from the *syce2* paternal genotype (top) and progeny from the *syce2* maternal genotype (bottom). Colored lines represent animals from 3 broods shown as colors in part A. (C) Examples of embryonic progeny of *syce2*^*+/+*^ mothers and normal-looking and malformed embryos from *syce2*^-/-^ mothers (top). Examples of metaphase chromosome spreads from embryonic cells stained with DAPI isolated from embryos from *syce2*^*+/+*^ mothers and normal-looking and malformed embryos from *syce2*^-/-^ mothers. The number of chromosomes in the spread is provided as n. Note, the embryos and spreads are independent and are not matched by animal. Quantification of the chromosome content in individual normal-looking (MUT-N) and malformed (MUT-D) offspring. Each column represents an individual embryo; dots represent the number of chromosomes in ten nuclei per embryo. Scale bar = 0.5 mm. (D) Number of eggs spawned and fertilization rate per cross for wild-type and *sycp1*^-/-^ mutants; paternal genotype (top) and maternal genotype (bottom). Analysis was performed as described in (A). *sycp1* paternal genotype includes Broods 2, 3, and 4. Brood 2 (S4297; blue): WT n = 4, MUT n = 3; Brood 3 (S4347; red): WT n = 3; MUT n = 2; Brood 4 (S44434; green): WT n = 2, MUT n = 4.The *sycp1* maternal genotype includes Broods 1, 2, and 4. Brood 1 (S3667; purple): WT n = 2, MUT n = 1; Brood 2 (S4297; blue): WT n = 1; MUT n = 1; Brood 4 (S44434; green): WT n = 8, MUT n = 8. Significance was determined using an unpaired t-test for pooled data. ns = not significant.; **** = p < 0.0001. (E) Percent Normal-looking offspring at 1- and 5-days post fertilization (dpf) from wild-type and *sycp1* maternal genotype. Colored lines represent animals from 3 broods in part D. (F) Analysis as in part C for progeny of *sycp1*^*+/+*^ and *sycp1*^*-/-*^ mothers. (G) Table showing a subset of embryos placed in the nursery from select broods and the percent that survived to adulthood.

In mice, mutations in *Syce2* and *Sycp1* lead to infertility due to a prophase I checkpoint that removes defective spermatocytes and oocytes [21]. We therefore tested whether fertility was affected in *syce2*^*-/-*^ and *sycp1*^*-/-*^ mutants. *syce2*^*-/-*^ males induce wild-type females to spawn similar numbers of eggs (272 ± 164) as wild-type males (234 ± 103) (Fig 4A), indicating normal mating behavior. The average fertilization rate of *syce2*^*-/-*^ males (74.6% ± 26.4%), while somewhat lower, was not significantly different from wild-type controls (90.4% ± 9.2%) (Fig 4A).

When crossed to wild-type males, *syce2*^*-/-*^ females also yield similar numbers of spawned eggs (183.6 ± 101.0) as wild-type females (207.6 ± 146.4) (Fig 4A). The average fertilization rate of *syce2*^*-/-*^ females (95.2% ± 4.8%), was also similar to wild-type controls (and 90.1% ± 25.7%) (Fig 4A). Remarkably, a high percentage of fertilized eggs from both *syce2*^*-/-*^ males and females appear morphologically healthy at 1- and 5-days post fertilization (dpf) (Fig 4B), though there was more variability among progeny of *syce2*^*-/-*^ mothers compared to males.

To determine whether the mutants produce euploid or aneuploid offspring, we counted metaphase chromosomes in cells (n = 10) isolated from 1-day old embryos of wild-type mothers, and both normal and malformed-looking embryos from *syce2*^*-/-*^ mothers. Both normal and malformed progeny from *syce2*^*-/-*^ females exhibit considerable variability in the number of chromosomes per nucleus (dots) within individual embryos (bars), while wild-type nuclei rarely deviate from 50 chromosomes (Fig 4C). These results indicate that defects in the meiotic machinery can lead to genome instability in the developing embryo leading to mosaic somatic aneuploidy.

Crosses involving *sycp1*^*-/-*^ males induced similar numbers of spawned eggs (258 ± 62) compared to wild-type controls (280 ± 1130) (Fig 4D). Similarly, spawning by *sycp1*^*-/-*^ females was not significantly different from controls (162 ± 87 and 178 ± 181) (Fig 4D). However, fertility is dramatically reduced in *sycp1*^*-/-*^ males (<0.04% ± 0.1%) compared to controls (75.9% ± 25%) while the fertility of *sycp1*^*-/-*^ females (82.8% ± 25.7%) is not (88.9% ± 13.5%) (Fig 4D). The majority of offspring from *sycp1*^*-/-*^ mutant females appear malformed at 1 and 5 dpf (Fig 4E). Over the course of this study, we found that 6 out of 4233 eggs (0.14%) were fertilized by *sycp1*^*-/-*^ males, indicating that completion of meiosis does occur at a low rate.

Similar to progeny of *syce2*^*-/-*^ mothers, a significant amount of mosaic somatic aneuploidy is observed among cells of embryos of *sycp1*^*-/-*^ mutant females (Fig 4F). These results demonstrate that the partial synapsis observed in *syce2*^*-/-*^ mutants is sufficient to produce fertile gametes and progeny that are visually normal at 1 and 5 dpf while complete asynapsis in the *sycp1*^*-/-*^ mutant primarily affects male fertility.

We raised a subset of normal-looking offspring from *syce2*^*-/-*^ and *sycp1*^*-/-*^ mothers past 5 dpf, and a substantial percentage survived into adulthood (Fig 4G). This was surprising given the severity of the synapsis defects, yet it suggested that chromosomes segregate normally at least in a subset of meiosis, that some degree of gamete aneuploidy is tolerated during development, or that aneuploid cells are preferentially removed or outcompeted by euploid cells during development to adulthood.

### Sex ratios are altered in *sycp1*^*-/-*^ mutants but not *syce2*^*-/-*^ mutants

**Fig 5:**
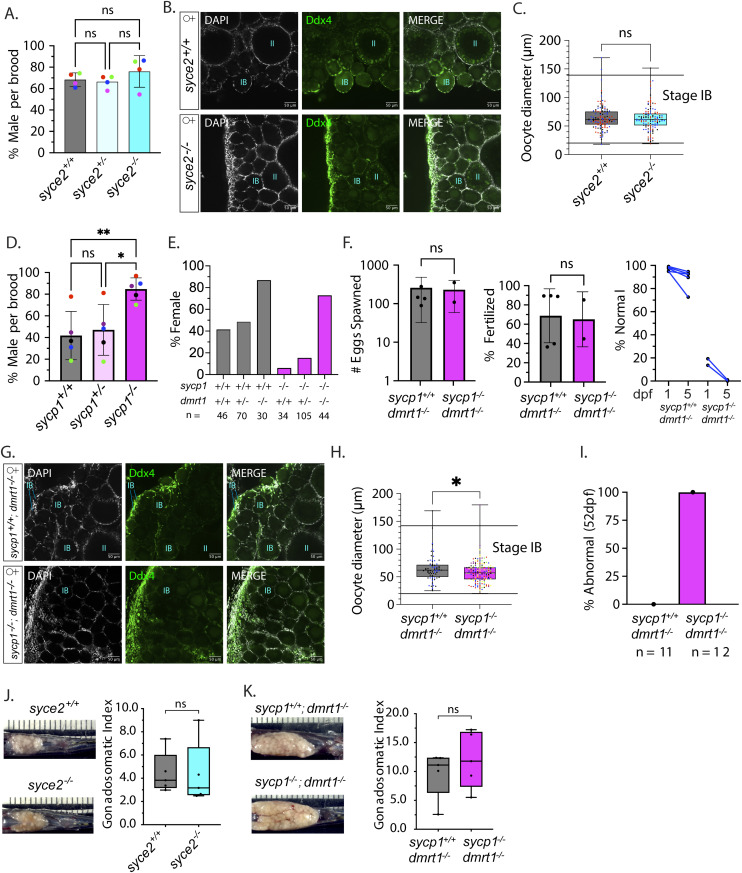
Normal sex ratios and gonad development in *syce2*^-/-^ mutants but not *sycp1*^*-/-*^ mutants. (A) Percentage of animals developing as male in *syce2*^*+/+*^, *syce2*^*+/-*^ and *syce2*^-/-^ genotypes from four independent experiments. Each data point indicates the percentage of males within each genotype out of total animals per brood: Blue (S4348) n= 99; Pink (S4461) = 342; Green (S4634) n = 318; Red (S4643) = 328. Significance was determined using repeated measures one-way ANOVA testing with Šidák’s multiple comparisons test; ns = not statistically significant. (B) Whole mount ovaries from 45 dpf *syce2*^+/+^ *and syce2*^-/-^ females imaged using confocal microscopy. Ovaries are stained for DAPI (gray) and Ddx4 (green). Scale bar = 50 µm. (C) Calculated diameters of individual Stage IB oocytes from 45 dpf ovaries *syce2*^+/+^ (n = 3; 118 total oocytes) and *syce2*^-/-^ (n = 3; 104 total oocytes). Different colors represent cells from different ovaries. Significance was determined using an unpaired t-test. ns = not statistically significant, * = p < 0.05. (D) Percentage of animals developing as male in *sycp1*^*+/+*^, *sycp1*^*+/-*^ and *sycp1*^*-/-*^ genotypes from five independent experiments. Each data point indicates the percentage of males within each genotype out of total animals per brood: Purple (S3667) n = 183; Blue (S4297) n= 237; Red (S4347) = 112; Black (S4395) n = 167; Green (S4434) n= 382. Significance was determined using repeated measures one-way ANOVA testing with Šidák’s multiple comparisons test; ns = not statistically significant, * = p < 0.05. (E) Percent females among genotypes of progeny from a *sycp1*^*-/+*^*; dmrt1*^*+/-*^ incross. n values are given for each genotype. (F) Number of eggs spawned by *sycp1*^*+/+*^*; dmrt1*^*-/-*^ (n = 5) *and sycp1*^*-/-*^*; dmrt1*^*-/-*^ (n = 2) females (left) and the percent fertilized by AB males (center). Significance was determined using an unpaired t-test. ns = not statistically significant, * = p < 0.05. Percent of normal progeny (right) at 1 and 5 dpf for each cross. (G) Whole mount ovaries from 45 dpf *sycp1*^+/+^ and *sycp1*^-/-^ females in a *dmrt1*^-/-^ background stained with DAPI (gray) and Ddx4 (green). Scale bar = 50 µm. (H) Calculated diameters of individual Stage IB oocytes from two 45 dpf *sycp1*^+/+^; *dmrt1*^-/-^ ovaries (n = 2; 81 total oocytes) and six 45 dpf *sycp1*^-/-^; *dmrt1*^-/-^ ovaries (n = 6; 151 total oocytes). Different colors represent cells from different ovaries. Unpaired t-test; Significance was determined using an unpaired t-test. **** = p < 0.0001. (I) Percent abnormal ovaries by size by visual inspection dissected from 52 dpf fish. *sycp1*^+/+^; *dmrt1*^-/-^ (n = 11) and *sycp1*^-/-^; *dmrt1*^-/-^ (n = 12). (J) Ovaries from adult *syce2*^*+/+*^ and *syce2*^*-/-*^ adult females (age = 11 months) *in situ* (left) and the gonadosomatic index of each (100 X gonad mass/total body mass) (right). N = 5 for each genotype. Unpaired t-test; ns = not statistically significant, * = p < 0.05. (K) Ovaries from *sycp1*^+/+^; *dmrt1*^-/-^ and *sycp1*^-/-^; *dmrt1*^-/-^ adult females (age = 13 months) as described in part J. N = 5 for each genotype. Unpaired t-test; ns = not statistically significant, * = p < 0.05. Note that the difference in ovary size from J is due to lower tank density and a greater amount of food per fish.

Sex determination in domesticated zebrafish is poorly understood, but it is not regulated solely by sex chromosomes [64]. Instead, all larvae (~14–20 dpf) regardless of their eventual sex produce early-stage oocytes (Stage IA) and those that produce a threshold number of oocytes continue to develop as females, while those that do not undergo oocyte apoptosis and develop as males [65]. Oocyte apoptosis in presumptive males initiates around 20–25 dpf and the gonad begins transitioning to a testis. By 30 dpf, most of the oocytes have been cleared from the gonad [66]. In addition, continuous oocyte production is required to maintain the female phenotype after sex determination [67]. Therefore, a male-biased mutant population can be indicative of a defect in oogenesis [58,68–72]. To gauge if sex ratios are altered in *syce2*^*-/-*^ populations, we measured the percentage of males in four different broods of each genotype. We found no significant difference in the percentage of males between *syce2*^*-/-*^ mutant (76.0% ± 14.8%) and wild-type (68.3% ± 6.4%) or heterozygous (66.4% ± 6.0%) populations, indicating that sufficient oocytes are present for female sex determination in the *syce2*^*-/-*^ mutant population (Fig 5A).

To test if oocytes appeared normal, we stained whole gonads from 45 dpf juveniles with an antibody to the germ cell marker Ddx4 and DAPI to visualize DNA. At this age, wild-type ovaries contain primarily Stage IA oocytes (leptotene to pachytene stages) in germline cysts with diameters ranging from ~8–20 µm and Stage IB oocytes (diplotene stage) that are surrounded by follicle cells (d = ~20–140 µm) and some Stage II oocytes (d = 140–340 µm) [73] are present. The overall morphology and distribution of the sizes of individual oocytes present in follicles was similar in *syce2*^*+/+*^ and *syce2*^*-/-*^ mutants (Fig 5B and 5C). There were very few Stage IB oocytes undergoing apoptosis in wild-type or mutants based on lack of nuclear fragmentation or other evidence of oocyte loss pointing to female-to-male sex reversal. Thus, oocyte development appears unaffected in *syce2*^*-/-*^ mutant females despite severe synapsis defects.

In contrast, the *sycp1*^*-/-*^ mutant population exhibited a significantly higher percentage of males (84.0% ± 11.7%) compared to wild-type (41.1% ± 25.6%) and heterozygous (45.7% ± 26.8%) populations (Fig 5D). These effects show that mutation of *sycp1*^*-/-*^ may be impacting oogenesis, but not to the extent of *sycp1*^*isa*/isa^ where a complete male population was reported [56]. The *sycp1* allele generated in our study encodes the first 97 amino acids of the protein compared to 408 amino acids encoded by the *sycp1*^*isa*^ allele [56]. While it is possible that the *isa* allele is disrupting meiosis in an unexpected way, chromosome spreads in both mutants show a complete absence of Sycp1 antibody staining. Given the genetic and environmental influences on zebrafish sex determination, it seems more likely that differences in animal husbandry and/or strain background (AB, this study, versus India) resulted in insufficient numbers of oocytes to support female sex determination or maintenance in the *sycp1*^*isa*/isa^ mutant that were analyzed in the previous study [56].

### Extending the sex-determination window by mutating *dmrt1* rescues the male bias of *sycp1*^*-/-*^ mutants

We reasoned that if the male bias of *sycp1*^*-/-*^ populations is due to insufficient oocyte production during the time sex is normally determined, then expanding the sex-determining window might increase the percentage of females. The sex-determining window depends on the relative timing of the initiation of meiotic prophase events and rate of apoptosis of early-stage oocytes, after which point the bipotential ovary transitions to a testis [65,68,74]. Mutation of the testis-promoting gene *dmrt1* reduces the number of these apoptotic oocytes and skews sex ratios to favor female development [75]. By contrast, a male-skewed population that is the result of checkpoint-mediated apoptosis in response to synapsis defects is not expected to be reversed by extending the sex-determining window due to an alternative apoptotic pathway. Instead, checkpoint-mediated apoptosis would lead to male development due to insufficient oocytes. We found that the *dmrt1* mutation dramatically increases the percentage of females in the *sycp1*^*-/-*^ background from 5.9% to 72.7% (Fig 5E). These results suggest that the male bias in *sycp1*^*-/-*^ mutants is likely due to the failure to create sufficient oocyte numbers for the determination or maintenance of the female sex in a subset of fish, rather than by oocyte loss through checkpoint-mediated apoptosis. Supporting this interpretation, we also found that *sycp1*^-/-^; *dmrt1*^-/-^ adult females spawned similar numbers of eggs and were fertilized at similar rates as *sycp1*^+/+^; *dmrt1*^-/-^ fish and embryo tracking shows similar decreased numbers of normal embryos similar to the *sycp1*^-/-^ single mutant (Figs 4F and 5F).

We next measured the areas of follicle oocytes in *sycp1*^-/-^; *dmrt1*^-/-^ 45 dpf fish as described above. We reasoned that a checkpoint-mediated delay, rather than checkpoint-mediated apoptosis, might provide an extended time to complete the chromosome events of meiotic prophase resulting in smaller oocytes. If DSBs result in apoptosis, we would expect to see loss of follicle oocytes and evidence of ovaries transitioning to testis morphology. We visually inspected stained whole gonads from 45 dpf juveniles with an antibody to the germ cell marker Ddx4 and DAPI to visualize DNA. Stage IB diplotene oocytes from *sycp1*^-/-^; *dmrt1*^-/-^ gonads were present near the edge of the ovary where Stage IA cells are located, without signs of fragmented DNA indicative of apoptotic nuclei or cells transitioning to testis (Fig 5G). This is in stark contrast to the *sycp1*^*isa*^ mutant where Stage IB oocytes fail to grow past 40 µm in diameter (~1300 µm^2^) in similarly aged animals [56]. On average, Stage IB oocytes in *sycp1*^-/-^; *dmrt1*^-/-^ females are somewhat smaller than in the *dmrt1*^-/-^ control (Fig 5H), however, the percent of oocytes greater than 1300 µm^2^ is similar in *sycp1*^-/-^; *dmrt1*^-/-^ and *dmrt1*^-/-^ ovaries (81%, n = 158 and 89%, n = 100, respectively). The smaller oocyte size could be due to gonads that start transitioning to testes, but without *dmrt1* revert to an ovary fate leading to female development [75]. Alternatively, cells could be transiting through prophase I somewhat slower in the *sycp1*^-/-^; *dmrt1*^-/-^ mutant. At 52 dpf, *sycp1*^+/+^; *dmrt1*^-/-^ and *sycp1*^-/-^; *dmrt1*^-/-^ ovaries were dissected for chromosome spreads. All ovaries from animals carrying the mutated *sycp1* allele were abnormally small (12/12) compared to those with the wild-type *sycp1* allele (0/11) (Fig 5I). However, analysis of homolog pairing and synapsis in *sycp1*^-/-^; *dmrt1*^-/-^ surface spread oocytes shows no difference in chromosome morphologies compared to *sycp1*^-/-^ oocytes (Fig 3E) and nuclei of all stages from leptotene to the P/D-like stage were recovered (S4 Fig).

In zebrafish mutants that undergo female-to-male sex reversal due to defects in oogenesis, the transition is often complete by 45–50 dpf [56,68,69,71,74]. Despite the normal fertility of *sycp1*^-/-^; *dmrt1*^-/-^ mutants, we wondered if the adult ovaries would also be smaller than the *sycp1*^+/+^; *dmrt1*^-/-^ control. For this we used visual inspection of adult ovaries of *syce2*^*-/-*^ and *sycp1*^+/+^; *dmrt1*^-/-^ and determined the gonadosomatic index (100 x gonad mass/total animal mass) of mutant and control adult ovaries. Since GSI can account for age and feeding regimen of age matched fish [76], we controlled age-matched fish raised in the same tanks for *syce2* and *sycp1*; *dmrt1* populations, respectively. The ovaries from *syce2*^*-/-*^ and *sycp1*^-/-^; *dmrt1*^-/-^ adult fish and the respective control animals from the same broods showed similar variation between individuals and the average GSIs were indistinguishable from controls (Figs 5J, 5K and S5A and S6A). These results show that while there may be some delay of follicle growth in the *sycp1*^-/-^; *dmrt1*^-/-^ juveniles, once female fate is established, the fertility and average GSI of adult animals with severe synapsis defects is indistinguishable from controls.

### Spermatogenesis is blocked in *sycp1*^*-/-*^ but not *syce2*^*-/-*^ mutants

**Fig 6:**
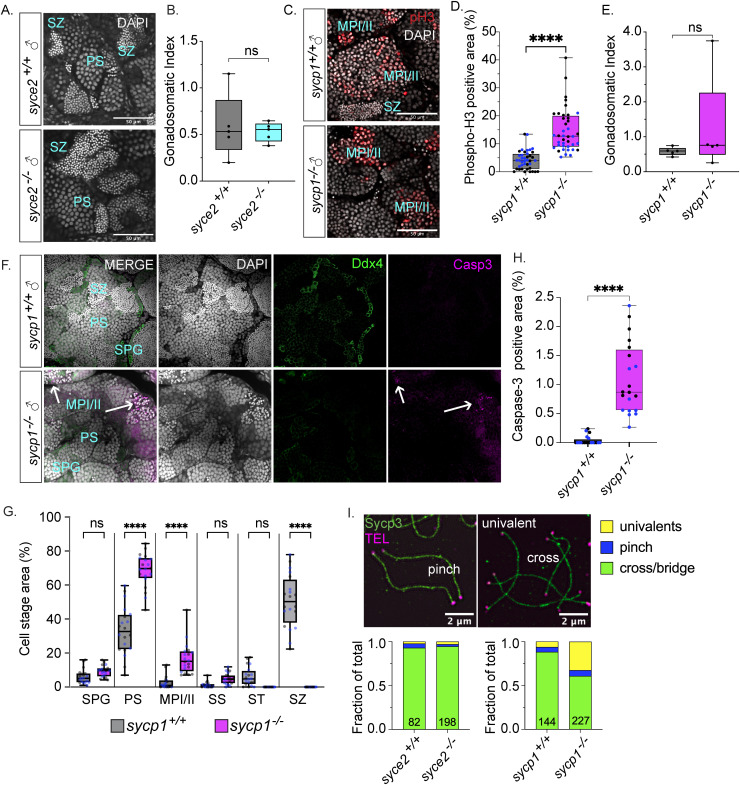
Spermatogenesis arrests at metaphase I/II in *sycp1*^*-/-*^ males but not *syce2*^*-/-*^ males. (A) Whole mount testes from adult (> 60 dpf) *syce2*^+/+^ and *syce2*^-/-^ males stained with DAPI (gray). Examples of primary spermatocytes (PS) and spermatozoa (SZ). Scale bar = 50 µm. (B) The gonadosomatic index of *syce2*^+/+^ (n = 5) and *syce2*^-/-^ (n = 5) testes. Unpaired t-test; ns = not statistically significant, * = p < 0.05. (C) Whole mount testes from adult (> 60 dpf) *sycp1*^+/+^ and *sycp1*^-/-^ males stained with DAPI (gray) and phospho-Histone H3 (pH3; red). Examples of spermatocytes in metaphase I or II (MPI/II) and spermatozoa are noted. Scale bar = 50 µm. (D) Percent of cyst area represented by phospho-histone H3 staining. Multiple image fields (n = 20) were analyzed each for wild-type *sycp1*^+/+^ testes (n = 2) and *sycp1*^-/-^ mutant testes (n = 2). Different colors represent data from same testis. Unpaired t-test; **** = p < 0.0001. (E) The gonadosomatic index of *sycp1*^+/+^ (n = 5) and *sycp1*^-/-^ (n = 5) testes. Unpaired t-test; ns = not statistically significant, * = p < 0.05. (F) Whole mount testes from adult (> 60 dpf) *sycp1*^+/+^ (top) and *sycp1*^-/-^ (bottom) males stained with DAPI (gray), Ddx4 (green) and cleaved caspase-3 (magenta). Arrows represent apoptotic nuclei. Scale bar = 50 µm. (G) Percent of cyst areas including spermatogonial cells (SPG), primary spermatocytes (PS), metaphase I/II (MPI/II), secondary spermatocytes (SS), spermatids (ST), and spermatozoa (SZ). Multiple image fields (n = 20) were analyzed each for wild-type *sycp1*^+/+^ testes (n = 2) and *sycp1*^-/-^ mutant testes (n = 2). Measurements from individual testes shown in gray or blue circles. One-way ANOVA testing with Šidák’s multiple comparisons; **** = p < 0.0001; ns = not statistically significant, * = p < 0.05. (H) Percent of cyst area with cleaved caspase-3 positive cells. Multiple image fields (n = 20) were analyzed each for wild-type *sycp1*^+/+^ testes (n = 2) and *sycp1*^-/-^ mutant testes (n = 2). Measurements from individual testes shown in black or blue circles. Unpaired t-test; **** = p < 0.0001. (I) Proportion of distinguishable chromosome configurations (cross/bridge bivalents, pinch bivalents and univalents) among diplotene (wild-type), pachytene-like (*syce2*^-/-^) and pachytene/diplotene-like (*sycp1*^-/-^) chromosomes. *syce2*^+/+^ (n = 82) and *syce2*^-/-^ (n = 198); Chi-squared test: ns = not significant, ns = p < 0.05. *sycp1*^-/-^ (n = 144) and *sycp1*^+/+^ (n = 227); Chi-squared p < 0.0001. Data is compiled from chromosomes spreads performed over seven independent experiments.

Next, we examined testes of *syce2*^*-/-*^ and *sycp1*^-/-^ males to test if spermatogenesis is disrupted in these mutants. Consistent with the normal fertility of *syce2*^*-/-*^ males, mutant testes have prominent sperm clusters and are morphologically indistinguishable from wild-type (Fig 6A). The GSI is similar in *syce2*^*-/-*^ and *syce2*^*+/+*^ males (Fig 6B) though there appeared to be somewhat higher asymmetry in the size of the left and right mutant testes (S4B Fig). By contrast, *sycp1*^-/-^ testes contain no sperm clusters indicating that spermatogenesis is incomplete (Fig 6C), yet there were several cysts containing irregularly shaped nuclei indicative of cells in metaphase I and metaphase II [77]. To test if these are cells in metaphase, we immunostained wild-type and *sycp1*^*-/-*^ testes with an antibody to the metaphase marker phospho-Histone H3 (Serine 10) [78]. We found that the irregularly shaped nuclei were positive for this marker, which accounted for ~15% of the total cyst area compared to ~4% for wild-type (Fig 6D). While *sycp1*^*-/-*^ testes were less opaque compared to wild-type due to lack of sperm, the GSI is similar to *sycp1*^*+/+*^ testes (Figs 6E and S6B).

The testis is made up of cysts where groups of cells are roughly in the same stage of spermatogenesis. We measured the relative cyst area for all germline cell types, including spermatogonial cells (SPG), primary spermatocytes (PS), metaphase I/II (MPI/II), secondary spermatocytes (SS), round spermatids (ST) and spermatozoa (SZ) and found a complete absence of round spermatids and spermatozoa (Figs 6F and 6G and S7) indicating that the primary defect in spermatogenesis in the *sycp1*^*-/-*^ mutant is not transit through meiosis I prophase, but instead by exiting metaphase I or metaphase II.

In mice, univalents cannot be stably oriented on the meiosis I spindle, which subsequently activates the spindle assembly checkpoint (SAC) and leads to apoptosis [79]. To test if apoptotic cells were present in the *sycp1*^*-/-*^ testes, we immunostained mutant and wild-type testes with an antibody to cleaved caspase-3, an indicator of apoptosis. Surprisingly, only ~1% of total cyst area was positive for this signal compared to the 15% positive for phospho-histone H3 marker, but this was significantly greater than 0.04% found in *sycp1*^*+/+*^ testes (Fig 6F and 6H). While it was difficult to stage these cells, they appeared to be enriched in clusters of cells that contained cells in metaphase II (S7 Fig).

### Univalents are more common in *sycp1*^*-/-*^ than *syce2*^*-/-*^ mutants

Next, we investigated whether the different reproductive outcomes in *syce2*^*-/-*^ and *sycp1*^-/-^ mutants could be due to the presence of univalents in *sycp1*^*-/-*^ mutants. We examined chromosome spreads from *syce2*^-/-^ and *sycp1*^*-/-*^ nuclei containing chromosomes with full-length axes and asynapsed diplotene chromosomes in wild-type spermatocytes. Because of the entangled nature of *syce2*^*-/-*^ and *sycp1*^-/-^ spread chromosomes, we limited our analysis to only those chromosomes where the full axes could be unambiguously traced end-to-end, which were generally on the outer edges of the spread chromosome region. We observed three types of chromosome configuration (Fig 6I): (1) bivalents in a pinched configuration, where the axes of sub-telomeric regions are co-aligned as parallel tracks but with no apparent crossing over of axes, suggestive of recombination-induced homolog co-alignment [48]; (2) bivalents with crossed or bridged axes, where axes overlap each other or appear bridged by a short stretch of Sycp3; or (3) univalents, where a full-length chromosome is found with no apparent partner. The proportion of univalents in *syce2*^*-/-*^ and *syce2*^*+/+*^ spermatocytes was comparable (3% vs 2.4%, respectively; Fig 6I). This result suggests that short stretches of synapsed-like regions observed in *syce2*^*-/-*^ mutants are sufficient to promote the formation of at least one crossover between each pair of homologs. By contrast, the proportion of univalents (but not pinched) in *sycp1*^*-/-*^ spermatocytes was higher than in *sycp1*^*+/+*^ (32.6% vs 6.3%; Fig 6C).

### Less efficient DSB repair in *syce2*^*-/-*^ and *sycp1*^*-/-*^ mutants

**Fig 7:**
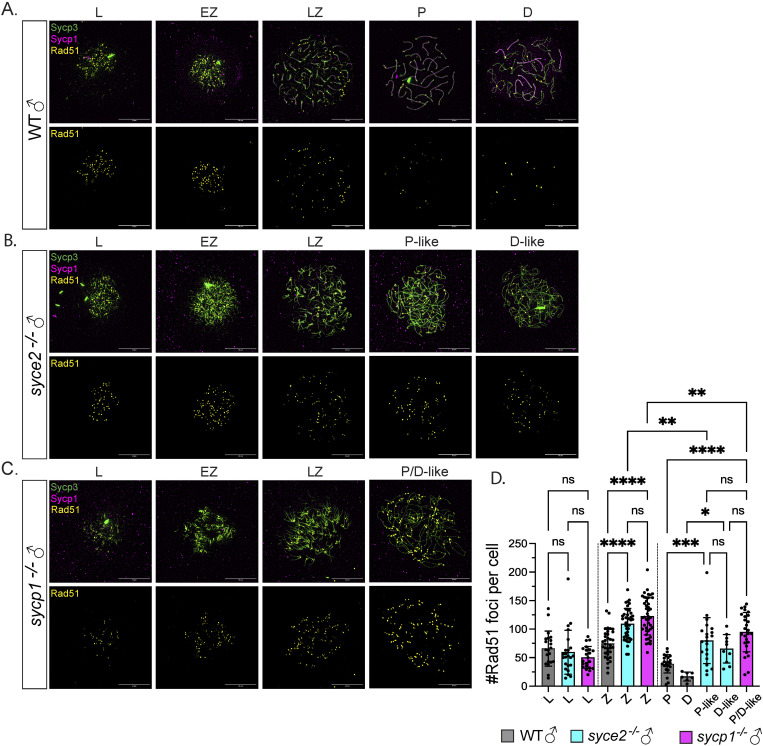
Inefficient DSB repair in *syce2*^-/-^ and *sycp1*^*-/-*^ mutants during prophase. (A–C) Surface-spread chromosomes from wild-type (A), *syce2*^*-/-*^ (B) and *sycp1*^-/-^ (C) spermatocytes immunostained for Sycp3 (green), Sycp1 (magenta) and Rad51 (yellow). Examples of spread chromosomes from meiotic prophase I: L, EZ, LZ, P, D, P-like (*syce2*^-/-^) and P/D-like(*sycp1*^-/-^). D-like nuclei (*syce2*^-/-^) are categorized as having full-length axes, but Sycp1 is largely absent from pseudo-synapsed regions throughout the nucleus. Scale bar = 10 µm. (D) Number of Rad51 foci per cell using data from (A–C). EZ to LZ nuclei are grouped together as zygotene (Z). Data is pooled from 2 independent experiments. n = 21, WT – L; n = 23, *syce2*^-/-^ – L; n = 22, *sycp1*^-/-^ – L; n = 34, WT – Z; n = 41, *syce2*^-/-^ – Z; n = 44, *sycp1*^-/-^ – Z; n = 22, WT – P; n = 7, WT – D; n = 21, *syce2*^-/-^ – P-like; n = 10, *syce2*^-/-^ – D-like; n = 26, *sycp1*^-/-^ – P/D-like. Significance was determined using ordinary one-way ANOVA testing with Šidák’s multiple comparisons test. ns = not significant; ** = p < 0.01; *** = p < 0.001; **** = p < 0.0001.

In mice, mutations in *Syce2 and Sycp1* lead to the accumulation of unrepaired DSBs [23,80]. We investigated whether there is a DSB repair defect in the analogous zebrafish mutants by examining spread spermatocyte chromosomes for the presence of Rad51, an indicator of unrepaired DSBs [81]. Although the pachytene-like and diplotene-like stages are indistinguishable in the *sycp1*^-/-^ spermatocyte spreads, we were able to identify diplotene-like spermatocytes in *syce2*^*-/-*^ mutants by finding nuclei in which the axes were full-length, but Sycp1 localization in synapsed-like regions is largely absent. In wild-type spermatocytes, Rad51 foci are present in leptotene (65.86 ± 30.6) and zygotene (74.53 ± 25.01) with a sharp reduction in pachytene (39 ± 16.02) and diplotene (16.86 ± 7.77) (Fig 7A and 7D). Both *syce2*^*-/-*^ and *sycp1*^-/-^ mutants have significantly more Rad51 foci from zygotene (109.3 ± 36.97 and 122.5 ± 32.54, respectively) to late prophase (79.86 ± 40.25, P-like; 65.5 ± 25.08, D-like; and 94.81 ± 34.43, P/D-like, respectively) (Fig 7A–D), indicative of increased DSB formation and/or less efficient DSB repair. The number of foci post-zygotene decreases in both mutants, suggesting that a fraction of DSBs are repaired (Fig 7D). Despite the synapsed-like nature of *syce2*^*-/-*^ homologs, there is no significant difference in Rad51 foci between *syce2*^*-/-*^ and *sycp1*^-/-^ mutants. We then immunostained whole *sycp1*^*-/-*^ mutant testes with an antibody to the DSB marker 𝛾H2AX, which allowed us to assess whether DSBs are still present in metaphase cells. We found that 𝛾H2AX is not detectable in metaphase nuclei, suggesting that despite their persistence through much of prophase I, DSBs are eventually repaired or the 𝛾H2AX marker itself is lost while DSBs persist (S8A Fig). We also immunostained whole-mount juvenile ovaries from 39 dpf *sycp1*^*-/-*^ mutant females. While 𝛾H2AX is observed in Stage IA oocytes that are in leptotene/zygotene as expected, 𝛾H2AX is not detectable in Stage IB oocytes that reach diplotene (S8B Fig), suggesting DSBs are repaired before oocytes enter the follicle stage.

### Hormad1 persists in partially synapsed *syce2*^*-/-*^ bivalents

**Fig 8:**
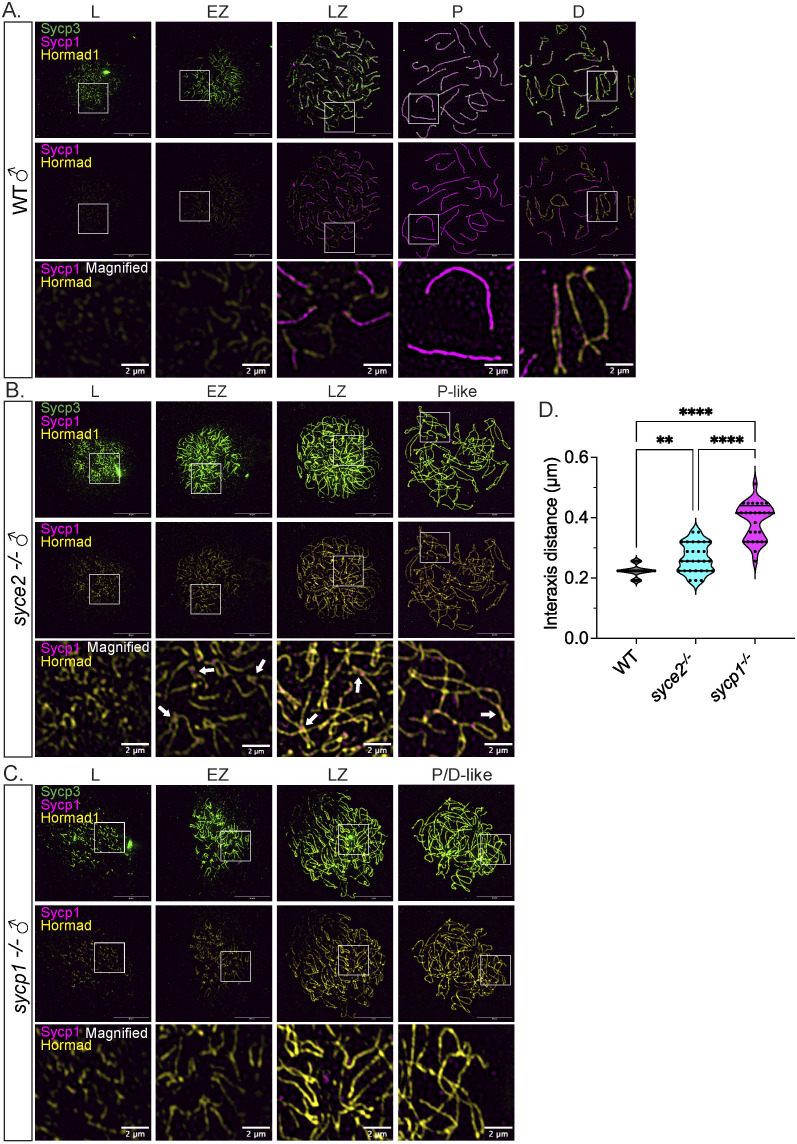
Sycp1 localization to synapsed-like regions is not sufficient for Hormad1 removal. (A–C) Surface-spread chromosomes from wild-type (A), *syce2*^*-/-*^ (B) and *sycp1*^-/-^ (C) spermatocytes immunostained for Sycp3 (green), Sycp1 (magenta) and Hormad1 (yellow). Examples of spread chromosomes from meiotic prophase I described in Fig 7A–C. Scale bar = 10 µm. Boxes represent magnified areas for each stage. Scale bar for magnified examples = 2 µm. Arrows represent Hormad1 localization to pseudo-synapsed regions with Sycp1. (D) Violin plot showing the distance between axes for co-aligned axial pairs in early zygotene for wild-type (n = 28), *syce2*^-/-^ (n = 28) and *sycp1*^-/-^ (n = 28) spermatocytes. 4 cells from each genotype were used for the analysis. Significance was determined using ordinary one-way ANOVA testing with uncorrected Fisher’s LSD test. ** = p < 0.01; *** = p < 0.001; **** = p < 0.0001.

In mice, DSB formation is promoted by HORMAD1, an axis-associated HORMA domain containing protein that is removed by TRIP13/Pch2 once a region has become synapsed [82]. In wild-type zebrafish, Hormad1 localization to meiotic chromosomes is transient since SC elongation immediately follows longitudinal growth of the axis. Consequently, only short stretches of Hormad1 staining are seen along asynapsed axes in spermatocytes during leptotene and zygotene [56] (Fig 8A). Hormad1 also binds to diplotene chromosomes once the synaptonemal complex is disassembled, similar to what has been observed in mice [83,84]. In *syce2*^-/-^ leptotene spermatocytes, Hormad1 localizes to asynapsed axes as seen in mouse (Fig 8B) [23]. Hormad1 persists on axial elements of partially synapsed regions in zygotene and then along full-length axes in pachytene-like *syce2*^-/-^ spermatocytes suggesting Syce2 may be required to remove Hormad1 from axes. We also found Hormad1 localization along axes in *sycp1*^-/-^ spermatocytes (Fig 8C), as was described previously for the *sycp1*^isa/isa^ mutant [56]. In mice, Hormad1 staining persists at centromeres of pachytene chromosomes, however, we saw no evidence of this localization on pachytene chromosomes in zebrafish spermatocytes. Together, these results suggest that meiotic chromosomes of *syce2*^*-/-*^ and *sycp1*^*-/-*^ spermatocytes lack an SC component that directs the removal of Hormad1.

Given the similarities in Hormad1 localization, DSB repair inefficiency and sub-telomeric co-alignment in *syce2*^*-/-*^ and *sycp1*^-/-^ mutants, we investigated whether the interaxis distance in the sub-telomeric region is also similar. Since the distance between axes can vary in spreads done on different days, we prepared a set of chromosome spreads from wild-type, *syce2*^-/-^ and *sycp1*^-/-^ spermatocytes from fish born and raised together. We focused our analysis on early- to mid-zygotene chromosomes when entanglement of chromosome axes is infrequent and found that the interaxis distance in *syce2*^*-/-*^ spermatocytes (0.266 ± 0.049) is intermediate between wild-type (0.224 ± 0.019) and *sycp1*^*-/-*^ (0.387 ± 0.062) (Fig 8D). These results suggest that Sycp1 contributes to close alignment of homologs even in the absence of a key central element protein.

## Discussion

Our study supports four main conclusions. First, SC components Syce2 and Sycp1 are required for full synapsis and efficient DSB repair during zebrafish meiosis. Second, the severity of meiotic disruption differs between the two mutants: Both *syce2*^*-/-*^ females and males are fertile, while *sycp1*^*-/-*^ females are fertile but produce more malformed progeny and males fail to produce sperm. Third, synapsis defects in these mutants do not appear to trigger a robust surveillance mechanism that blocks progression through meiotic prophase I. Fourth, maternal SC mutations cause embryonic genome instability, as shown by somatic mosaic aneuploidy in progeny.

Our finding that chromosome ends are co-aligned while in the bouquet in *sycp1*^*-/-*^ mutants is consistent with previous findings [56]. When the axes reach full length, the majority of co-alignment is disrupted, but not more or less than what is seen for desynapsed bivalents of wild-type diplotene chromosomes. If co-alignment in leptotene nuclei is mediated by recombination intermediates [48], then these appear to be resolved in the mutant by the time the axes achieve their full length. Crossed axes are evidence that crossing over has occurred, suggesting that *sycp1*^*-/-*^ spermatocytes have reached the diplotene-like stage of prophase, however, a significant increase in univalents is observed suggesting that at least a subset of recombination intermediates are repaired as noncrossovers [56]. A similar progression is seen in *syce2*^*-/-*^ mutants except that in the intervening period between zygotene and pachytene stages, short stretches of synaptonemal complex have formed and then disassembled. In both mutants, Hormad1 protein is bound along the lengths of axes from throughout prophase I. These events are summarized in S9 Fig. It appears that crossing over may be more efficient in the *syce2*^*-/-*^ mutant since fewer univalent are observed in chromosome spreads. Nonetheless, a high degree of somatic mosaic aneuploidy is seen in both normal and malformed embryos within 24 hours suggesting gamete aneuploidy. The presence of univalents in the *sycp1*^*-/-*^ mutant may account for the high percentage of malformed progeny of females and also male sterility.

We found that *syce2*^*-/-*^ and *sycp1*^*-/-*^ females produce normal numbers of fertile eggs and some progeny that can live to adulthood despite extensive asynapsis and inefficient DSB repair during meiotic prophase I. This is in striking contrast to what is seen in mice, where *Sycp1* mutant females along with other central element mutants exhibiting synapsis defects are infertile and oocytes undergo apoptosis before reaching the follicle stage in diplotene [80,85–87]. Though, *Sycp2*^*-/-*^ and *Sycp3*^*-/-*^ female mice with interrupted synapsis due to defects in axial element structures are sub-fertile and produce aneuploid oocytes [88,89]. Ovaries of 45 dpf zebrafish from *syce2*^*-/-*^ and *sycp1*^*-/-*^ females contain large numbers of Stage IB oocytes at the follicle stage with no evidence of loss or apoptotic bodies or any loss of oocytes that are characteristics of female-to-male sex reversal at this age. The gonadosomatic indices of adult *syce2*^*-/-*^and *sycp1*^*-/-*^ females are similar to controls. Together, these results suggest that zebrafish females tolerate asynapsis and are fertile whereas in mice asynapsis leads to checkpoint-mediated apoptosis and infertility. Instead, an apparently weaker, or absent, surveillance system monitoring synapsis defects in zebrafish appears to be more similar to budding yeast, *Drosophila*, and *Arabidopsis*, which form meiotic spores or gametes despite extensive asynapsis [30,90]. While we cannot rule out that a subset of oocytes are undergoing checkpoint-mediated apoptosis during the leptotene to pachytene stages as seen in *C. elegans* [36], zebrafish females do not display reduced egg-laying capacity observed in *syp-1* null mutant worms [38,39]. We also cannot entirely rule out that *syce2*^*-/-*^ and *sycp1*^*-/-*^ zebrafish mutants lack a synapsis checkpoint-inducing signal as we posited for the *spo11*^*-/-*^mutant that lacks meiotically induced DSBs and synapsis [48]. Though we think it unlikely, we also cannot rule out that the syce2^-/-^ mutant produces a nonconserved 88 amino acid protein fragment that interferes with a potential checkpoint activity.

In mice, prophase I arrest in response to unrepaired DSBs or asynapsis is coupled to a cellular program leading to meiotic silencing of unpaired chromatin (MSUC) [91–93]. MSUC is a process related to meiotic sex chromosome inactivation (MSCI) that silences unpaired regions of sex chromosomes in the heterogametic sex to evade checkpoint activation [91,92,94]. Similarly, the unpaired X chromosome in *C. elegans* is also transcriptionally silenced to evade checkpoint signaling in the male germline [95–98]. Evidence of MSUC or MCSI has not been described in fish species, though self-pairing of folded supernumerary B chromosomes and nonhomologous synapsis of sex chromosomes may play a role in circumventing a MSUC activation [99–101]. However, evidence of both male and female heterogamety (e.g., XY and ZW) implies switching from one system to another in the teleost lineage (e.g., genera *Oryzias* [102] and *Xiphophorus* [103]) suggesting asynapsis may be more generally tolerated. In zebrafish, fertile triploid females can be generated in a *cntd1* mutant background, suggesting the presence of 25 unpaired chromosomes does not inhibit female development.

Teleosts make up the most species-rich clade among vertebrates with over 30,000 species [104]. Accommodation of asynapsis in this lineage could be a vestige of the whole genome duplication event that occurred 320–350 million years ago giving rise to a common polyploid ancestor [105]. Moreover, more recent genome doubling (autoploidy) and hybridization between different species (allopolyploidy) is common among fish species [106]. Polyploidy could create challenges for meiotic homolog pairing and recombination and these challenges might be better tolerated without a stringent checkpoint response to asynapsis [107]. Polyploidy and whole genome duplication have occurred multiple times in plants, often contributing to speciation [108]. Polyploidization across plant species is often attributed to relaxed meiotic checkpoints [107]. *Arabidopsis* mutants with asynapsis and unrepaired DSBs complete meiosis, suggesting a weak synapsis and/or recombination checkpoint [109]. Though, at elevated temperatures a specialized variation of the prophase I checkpoint appears to exist in *Arabidopsis* [110]. Among non-mammalian vertebrate species, polyploidy is most common in amphibians and fishes, [111] but no undisputed evidence of a mammalian polyploid species exists [112]. It has also been suggested that reduced checkpoint stringency could favor animal taxa that produce large numbers of progeny in fresh water conditions where fertilization occurs outside of the body [113,114].

All zebrafish lacking germ cells develop as males and have testes [115]. Several zebrafish mutants with defects in meiotic processes develop solely as males, including those with mutations in chromosome axis genes s*ycp2* [70] and *sycp3* [74], the meiosis-specific cohesin subunits *smc1b* (ortholog of human *SMC1β)* [116] and *rad21l1* (ortholog of human *RAD21L*) [71], and the DSB repair genes *brca2* [69,72], *rad51* [117] and *fancl* [68] and other Fanconi anemia genes [58] exhibiting a range of phenotypes from shortened axial structures, reduced DSB formation, unrepaired DSBs, and asynapsis. The lack of females is partially rescued in *sycp3*, *rad21l1*, *brca2*, *rad51,* and *fancl* mutants by reducing TP53-mediated apoptosis [68,69,71,72,74,117] consistent with observations in mice indicating that the TP53 ortholog is involved in eliminating cells with unrepaired DSBs [118]. While we did not directly examine the role of the TP53 ortholog, our findings are not consistent with significant oocyte removal by checkpoint-mediated apoptosis in the mutants examined in this study since increasing the window of sex determination allows for the vast majority of *sycp1*^*-/-*^*; dmrt1*^*-/-*^ juveniles to achieve female-sex fate and grow to be fertile adults. The alternative outcome was not observed, where checkpoint-mediated apoptosis causes *sycp1*^*-/-*^*; dmrt1*^*-/-*^ animals to develop as infertile males. We predict that mutating *dmrt1* and prolonging the sex determination window would also suppress female-to-male sex reversal phenotype of the *sycp1*^*isa/isa*^ mutant, and possibly other zebrafish mutants, including *fancl* and *rad21l1* mutants that have been shown to produce Stage IB oocytes before undergoing female-to-male sex reversal [56,68,71]. The smaller oocytes seen in *sycp1*^*-/-*^; *dmrt1*^*-/-*^ double mutants compared to *dmrt1*^*-/-*^ single mutants may indicate that progression through early meiotic prophase I (e.g., leptotene through pachytene) is slower in the absence of *sycp1*, perhaps due to slower pairing kinetics, removal of entanglements, or delayed DSB repair that might transiently activate a DNA repair checkpoint that functions during prophase I. While chromosome spreads of double mutant oocytes show all stages of meiotic prophase I f(leptotene through pachytene/diplotene-like stages), our experiments do not rule out that at least a subset of cells in early prophase I are undergoing checkpoint-mediated apoptosis. Notably, we see a similar progression through the chromosome events of prophase I in males and no evidence of prophase I checkpoint-mediated apoptosis by cleaved-caspase 3 staining in primary spermatocytes.

The dramatic increase in metaphase spermatocytes in *sycp1*^*-/-*^ mutant males suggests that cells can transit prophase I in the absence of synapsis but the increased level of univalents, or perhaps chromosome entanglements, are causing cells to arrest in MI or in some cases MII. Zebrafish *cntd1* and *mlh1* mutant spermatocytes reach the pachytene stage with fully synapsed chromosomes but are defective in forming crossovers. In these cases, spermatocytes arrest at metaphase I, triggering apoptosis [119,120], possibly mediated by the spindle assembly checkpoint (SAC). Other evidence for the activation of the SAC during zebrafish spermatogenesis comes from studies showing metaphase arrest and apoptosis in testes treated with nocodazole at an elevated temperature; in this case, apoptosis is suppressed by mutation of the SAC checkpoint kinase gene *mps1 (ttk)* [121]. Interestingly though, we found that only about 1% of the total cyst area measured in *sycp1*^*-/-*^ testes was positive for the apoptotic marker cleaved caspase 3 despite ~15% of cells being arrested in metaphase I or II. It is possible that there is an insufficient number of univalents in the majority of metaphase I cells to activate the SAC in *sycp1*^*-/-*^ mutants and instead, spermatogenesis is blocked by a SAC-independent mechanism.

Finally, one aspect of our work that was surprising was the high degree of somatic mosaic aneuploidy observed among cells of individual embryos of *syce2*^*-/-*^ and *sycp1*^*-/-*^ mothers. These events suggest that zebrafish can tolerate high levels of aneuploidy during early development and also suggests that errors in meiosis can promote genome instability in progeny.

## Materials and methods

### Ethics statement

The University of California Davis Institutional Animal Care and Use Committee (IACUC) has approved of this work under the protocol #23636. For noninvasive procedures (e.g., fin clips for genotyping), zebrafish were anesthetized using tricaine. Invasive surgical methods were performed on fish euthanized by submerging fish in ice water.

### Zebrafish husbandry

Zebrafish husbandry was performed as previously described [122] with the following modifications. At 5 days post fertilization (dpf), zebrafish larvae were placed into 1 L aquaria at 40 fish/tank. At 5–10 dpf larvae were fed ~ 3 mL rotifers *ad libitum*. From 10–14 dpf larvae were fed 3 mL rotifers *ad libitum* and 1 drop concentrated brine shrimp twice daily. From 14–23 dpf, larvae were fed 2 drops concentrated brine shrimp twice daily. From 23–30 dpf, larvae were fed 3 drops concentrated brine shrimp twice daily. At 30 dpf, juvenile fish were moved to 9 L tanks continuous system water flow and no more than 40 fish. From 30–60 dpf, juveniles were fed ~ 500 µL concentrated brine shrimp and 100 mg Gemma Micro 300 twice daily. Starting at 60 dpf, fish were fed 100 mg Zebrafish Select Diet twice daily.

### Mutant generation and identification

The wild-type AB strain was used to generate the *syce2*^*-/-*^ and *sycp1*^*-/-*^ mutants. All fish used in the experiments were outcrossed to the AB strain. The *syce2^-/-^* and *sycp1^-/-^* mutants were generated with CRISPR-Cas9 using the CRISPR Design Tool (Synthego, Redwood City, CA, USA). gRNA target sequences: 5’ UUCACCAGCCAACAAUACAG 3’ (*syce2*, exon 4) and 5’ CGAGCAGUUUGGAGUACAGC 3’ (*sycp1*, exon 5). One-cell embryos were injected with 300 ng/µL gRNA, 2µM Cas9-NLS purified protein (MacroLab, University of California, Berkeley), and phenol red (5% in 2M KCl). Injected founder fish were raised to adulthood and outcrossed to wild-type fish. Genomic DNA was extracted from the caudal fin of resulting offspring to screen for mutations via High Resolution Melt Analysis (HRMA) [48]. *syce2* HRMA primers: forward 5’-TGATGATTCAGGAATTGGTGTT-3’; reverse 5’-CATCTATTGGTGAATTGTTTGGAG-3’. *sycp1* HRMA primers: forward 5’-GGAGCTTTTTGTTGTTGTTGCAT-3’; reverse 5’-ATCGGTTCTGAACTTCCAGAGT 3’. Mutations were identified in offspring of *syce2* injected fish using PolyPeak Parser [123] after Sanger Sequencing of PCR products amplified by Phusion DNA polymerase (New England Biolabs catalog #M0530L). Mutations were confirmed by sequencing PCR products from homozygous mutants. PCR primers: forward 5’-ACCATTTCGTTTCTTGTGTCAGT-3’; reverse 5’-ACACGTTTTACTAATCTGCCTGT-3’. Mutations were identified in offspring of *sycp1* injected fish using PCR products amplified by Phusion DNA polymerase and cloned into the pCR Blunt II-TOPO vector (Invitrogen catalog #450245). *sycp1* PCR primers: forward 5’-GCCAATGGAAAAGGAAGAGGTC-3’; reverse 5’-TGCTTAGACTTTCATTTGCGAACT-3’. The *dmrt1* mutation was generated as previously described [75].

## Genotyping

*syce2* and *sycp1* fish were genotyped using the HRMA primers above. *dmrt1* HRMA primers (BWD934 and BWD935) are reported elsewhere [75].

### Protein alignment

Protein alignments were done using the Clustal Omega multiple sequence alignment tool [124] using protein sequences from the UniProtKB database for zebrafish, human, and mouse Syce2 orthologs (Q56P19, Q6PIF2 and Q505B8, respectively) and Sycp1 orthologs (F1QYS1, Q15431 and Q62209, respectively).

### RT-PCR

Total RNA extraction from individual testes was performed as previously described [71]. Total RNA extraction from individual adult (> 60 dpf) testes was performed as previously described with the following modification: upon liquid phase separation 70 µL of the top, clear aqueous solution was purified using the RNA Clean and Concentrator-5 Kit (Zymo Research catalog #R1015). *syce2* RT-PCR primers: forward 5’-CATACATCAGCAAAGGTGCCAG-3’; reverse 5’-CACCAAAAGGTTCCAGGGCT-3’. *sycp1* RT-PCR primers: forward 5’-CGTCGAGAAGCTTTGGTGACT-3’; 5’-TGCCACCATCCTCTGAACAT-3’. *eef1a1l1* (control) RT-PCR primers: forward 5’-CTACCTACCCTCCTCTTGGTCG-3’; reverse 5’-CCTTAAGTAGAGTGCCCAGGT-3’.

### Sex ratios

The sex of adult zebrafish (> 60 dpf) was determined by the absence or presence of an ovipositor found only in females. For juvenile animals between ~45–52 dpf, the sex was determined by the presence of Stage IB oocytes in the follicle stage.

### Chromosome spread preparation and staining

Four to seven testes from adult males (> 60 dpf) were dissected and prepared as previously described in [48] with minor modifications in reagents used: Trypsin inhibitor (Sigma-Aldrich, catalog #T9253); Paraformaldehyde (ThermoFisher, catalog # 28908). Five to fifteen ovaries were dissected from juvenile females (46–52 dpf) and prepared as previously described [48].

PNA telomere probe and primary and secondary antibody staining was performed as previously described [48]. PNA Telomere probe used: TelC-Alexa647 (PNA Bio Inc, catalog #F1013). Primary antibodies used: 1:200 rabbit anti-Sycp3 (Abcam, catalog #ab150292, discontinued); 1:200 rabbit anti-Sycp3 (Novus Biologicals, catalog #NB300232); 1:100 chicken anti-Sycp1 [48]; 1:100 mouse anti-Rad51 (ThermoFisher catalog# MA5–14419, discontinued); 1:100 mouse anti-Hormad1 [56]. Our attempts to probe for crossovers in wild-type meiotic nuclei with commercially available and a custom made anti-Mlh1 antibody were unsuccessful. Secondary antibodies used at 1:1000: goat anti-rabbit IgG Alexa Fluor 488 (ThermoFisher, catalog #A-11008); goat anti-chicken IgY Alexa Fluor 568 (ThermoFisher, catalog #A-11041); goat anti-chicken IgY Alexa Fluor 594 (ThermoFisher, catalog #A-11042); goat anti-mouse IgG Alexa Fluor 647 (ThermoFisher, catalog #A-21236). Slides not stained for telomeres were rehydrated in ~500 µL 1X PBS and then proceeded to primary antibody staining. Slides were mounted with Prolong diamond antifade mountant with DAPI (ThermoFisher, catalog #P36966), Prolong diamond antifade mountant (ThermoFisher, catalog #P36970) or Prolong glass antifade mountant with NucBlue (ThermoFisher, catalog #P36985).

### Whole mount gonad and staining

Adult testes (> 60 dpf) were dissected and stained as previously described [48]. Juvenile ovaries (39–52 dpf) were dissected and stained similarly to adult testes. Primary antibodies used: 1:2000 chicken anti-Ddx4 [48]; 1:200 mouse anti-pH3 (Abcam, catalog #ab14955); 1:100 rabbit anti-𝛾H2AX [125]; 1:200 rabbit anti-𝛾H2AX (GeneTex, GTX127342) rabbit anti-cleaved Caspase-3 (Asp175) (Cell Signaling Technology, catalog #9661S). Secondary antibodies used at 1:300: goat anti-chicken IgY Alexa Fluor 488 (ThermoFisher, catalog #A-11039); goat anti-mouse IgG Alexa Fluor 594 (ThermoFisher, catalog #A-11032); goat anti-rabbit IgG Alexa Fluor 594 (ThermoFisher, catalog #A-11012).

### Metaphase spreads

Embryos at ~19–32 hours post fertilization were manually dechorionated and prepared according to [126] with the following modifications: Embryos were incubated in colchicine for 2 hours 15 minutes. The yolk was removed within 16 minutes followed by incubating on ice for 16 minutes. Embryos were mechanically homogenized for 3 minutes in 50 μL of fixative solution followed by brief centrifugation for 1 minute. Instead of pre-equilibrating slides to 4 ºC, slides were held face down immediately above a 55°C water bath for 15–20 seconds. The entire cell suspension from one embryo was then dropped onto the center of the slide held at a 30–45° angle to allow the cell suspension to spread. The slide was held face down above a 55°C water bath for 15–20 seconds, air dried for 2 minutes, passed back and forth over a flame 5 times and washed in a coplin jar with 1X PBS for 5 minutes. Slides were air dried or excess PBS was removed with a nasal aspirator to facilitate drying. Slides were stained with Vectashield antifade mounting medium with DAPI (Vector Laboratories, catalog #H-1200) and covered with a glass coverslip.

### Imaging

Images of stained meiotic chromosomes and gonads were collected at the Department of Molecular and Cellular Biology Light Microscopy Imaging Facility at UC Davis. Chromosomes spreads were imaged using the Nikon N-SIM Super-Resolution microscope in 3D-SIM imaging mode with APO TIRF 100X oil lens. Images were collected and reconstructed using the NIS-Elements Imaging Software. Gonads were imaged using the Zeiss 980 Laser Scanning Microscope with Airyscan using LMS confocal. Gonads immunostained for 𝛾H2AX were imaged using the Olympus FV1000 laser scanning confocal microscope. Images of metaphase chromosomes were collected on a Zeiss MetaSystems Imager Z2 microscope 63X lens with MetaFer 5.

Cleaved caspase-3 and phospho-histone H3 staining in testes was imaged using Andor BC43 CF spinning disk confocal microscope. All images were processed using Fiji ImageJ software. Only linear modifications to brightness and contrast of whole images were applied.

### Quantification of mean synapsed length and Sycp1 fluorescence intensity

Mean synapsed lengths per cell were determined by tracing stretches of Sycp1 lines using the segmented line tool in ImageJ. Since Sycp1 staining is often discontinuous between co-aligned axes in *syce2* mutants, we included regions where co-aligned axes were in a partially synapsed-like configuration (i.e., separated by ~0.2 µm). To calculate the mean fluorescence intensity of Sycp1 signal per cell, we subtracted a mean fluorescence intensity of six boxes taken from the background region of each spread from the mean fluorescence intensity of each scanned Sycp1 segment from the same spread. The average of the adjusted means for each nucleus was normalized to the value calculated from the wild-type EZ spreads to get the relative levels for mutant and wild-type at each stage.

### Interaxis distance

The interaxis distance was measured by drawing a line perpendicular to parallel co-aligned axes in ImageJ to generate kymographs. We identified the maximum intensity signals from the kymographs to determine x-values of the two highest peaks. The x-values were subtracted to calculate the interaxis distance.

### Number of co-aligned sub-telomeric regions

Axes were considered co-aligned if parallel regions of the axes were separated by less than 0.5 µm. In wild-type spermatocytes, synapsed regions were included as co-aligned. Slightly misaligned axes in otherwise parallel axes were considered as co-aligned since the orientation of the chromosomes may shift when the 3D nucleus is flattened to 2D. Axes that were perpendicular to each other but had no other parallel region between them were not considered co-aligned. All instances of co-aligned axes of asynapsed chromosomes ends were assessed independently since the high levels of chromosome entanglement in the *sycp1*^*-/-*^ mutant made it difficult to assess if one or both ends were co-aligned.

### Fertility testing and embryo tracking

To analyze fertility, individual mutant fish were placed in a divided mating tank overnight with one or two AB wild-type fish of the opposite sex. The divider was removed soon after the onset of light, and any eggs produced were collected with a strainer and categorized as fertilized, unfertilized or decomposed. Fertilized eggs were placed in a petri dish at 28–30 °C with embryo media and monitored at 1 and 5 dpf for malformities.

### Oocyte area

The segmented line tool of ImageJ was used to trace an outline of individual diplotene oocytes in single optical sections in intact gonads imaged using the Zeiss 980 Laser Scanning Microscope. Ovaries were imaged with one edge in the field of view for comparison between images. The area is determined by the number of pixels bound by the perimeter and the pixel density of the image.

### Caspase and metaphase area

Cells showing cleaved Caspase-3 or phospho-Histone H3 signal were traced using the ImageJ freehand selection tool and their areas were measured. The relative areas were calculated by taking (area of cells with signal/total area of meiotic cells) x 100%. Cells were staged according to [77]. For S7 Fig four images were stitched using the Grid/Collection stitching plugin in ImageJ [127].

### Gonadosomatic index

Whole fish and dissected gonads were weighed and the gonadosomatic index was calculated by taking (gonad weight/whole fish weight) x 100%. Fish were not fed the morning of the analysis.

### Statistics

All statistical analyses were performed using GraphPad Prism 10. Statistical tests are reported in the figure legends. Comparisons between controls and mutants for individual experiments were done using fish born on the same day.

## Supporting information

S1 Fig Targeted mutation of *syce2* and *sycp1* via CRISPR/Cas9. (A) Creation of the *syce2* mutation by CRISPR/Cas9 showing a 2 nucleotide (nt) deletion within exon 4 (out of 5). The CRISPR target site (green) and premature stop codon (yellow) are shown. (B) Creation of the *sycp1* mutation by CRISPR-Cas9 showing the site of the mutation within exon 5 (out of 32). The CRISPR target site (green), premature stop codon (yellow) and the complex mutation (orange) are shown. The complex mutation leads to a net increase of 22 nt. (C–D) RT-PCR from wild-type, *syce2*^-/-^ and *sycp1*^-/-^ testes. Two biological samples were used for each genotype. -RT = no reverse transcriptase. *eef1a1l1* is the positive control. (D) Note the slower migration in the *sycp1*^-/-^ samples due to the complex mutation with a net increase of 22 nt.
(PDF)

S2 Fig EZ and LZ stages from *syce2*^+*/*+^ and *syce2*^*-/-*^ spermatocytes. Magnified images from EZ and LZ stages from Fig 2A and 2B.
(PDF)

S3 Fig Identification of wild-type diplotene spermatocytes. Examples of diplotene spermatocytes from *sycp1*^+/+^ males stained for Sycp3 (green), Sycp1 (magenta and gray) and Telomeres (cyan). Spermatocytes were classified as diplotene if they contained three or more bivalents with 50% or more desynapsis. Arrows point to examples of desynapsed sub-telomeric regions that were analyzed for co-alignment (< 0.5 µm). Scale bar = 10 µm.
(PDF)

S4 Fig *dmrt1* mutation does not affect chromosome co-alignment. (A–B) Surface-spread chromosomes from *sycp1*^+/+^; *dmrt1*^-/-^ (A) and *sycp1*^-/-^; *dmrt1*^-/-^ (B) oocytes stained for Sycp3 (green) and Sycp1 (magenta and gray). Examples of spread chromosomes from meiotic prophase described in Fig 3 A–B. Scale bar = 10 µm.
(PDF)

S5 Fig Ovaries and testes from *syce2*^+*/*+^ and *syce2*^*-/-*^ animals. Brightfield images of ovaries (A) and testes (B) *in situ* that were used to calculate the gonadosomatic index (GSI) for each sex.
(PDF)

S6 Fig Ovaries and testes from *sycp1*^+*/*+^ and *sycp1*^*-/-*^ animals. Brightfield images of ovaries (A) and testes (B) *in situ* that were used to calculate the gonadosomatic index (GSI) for each sex. Graphs are the same as in Fig S5.
(PDF)

S7 Fig Cleaved caspase-3 positive cells in a *sycp1*^*-/-*^ testis. Four stitched images showing the germ cell landscape of a *sycp1*^*-/-*^ testis with examples of cleaved Caspase-3 stained cells (arrows). (A) Testis region stained with DAPI (cyan), cleaved Caspase-3 (magenta), and Ddx4 (green). Patches of cells in metaphase I or metaphase II outlined in yellow. A subset of cells in the outlined regions are positive for cleaved Caspase-3 staining. (B) Larger field image showing the location of the cells in the testis in part A. (C) Same panel as in part B showing DAPI (gray). (D) Same panel as in part B showing cleaved Caspase-3 (magenta). (E) Same panel as part B showing Ddx4 (green).
(PDF)

S8 Fig yH2AX staining in *sycp1*^*-/-*^ testes and oocytes (A) Whole mount testes from adult (> 60 dpf) *sycp1*^+/+^ and *sycp1*^-/-^ males stained with DAPI (gray) and 𝛾H2AX (green). Dashed lines represent metaphase nuclei. Scale bar = 50 µm. (B) Whole mount ovaries from 39 dpf *sycp1*^+/+^ and *sycp1*^-/-^ females stained with DAPI (gray) and 𝛾H2AX (green). Examples of Stage IA oocytes (leptotene/zygotene stage) and Stage IB oocytes are indicated by blue lines. Scale bar = 50 µm.
(PDF)

S9 Fig Model of chromosome events during meiotic prophase. (A–C) Temporal progression of chromosome axis formation, DSB repair, Hormad1 localization, synapsis and homolog juxtaposition. (A) In wild-type zebrafish, DSBs form in early meiotic prophase I and are gradually repaired. Hormad1 localizes to asynapsed regions and is displaced upon synapsis. As synapsis progresses, homologs are “zippered up”. (B) In *syce2*^*-/-*^ mutants, there is partial recruitment of Sycp1 to synapsed-like regions and unaligned axes. Hormad1 localizes along the entire length of chromosomes. DSBs form but repair is less efficient. Without a fully formed SC, chromosome co-alignment is limited to sub-telomeric regions. (C) In *sycp1*^*-/-*^ mutants where there is no synapsis, Hormad1 localizes along entire axes and DSB repair is less efficient. Chromosome co-alignment is also limited to sub-telomeric regions. Not shown are other central element components: Syce1, Syce3, Six6os1, and Tex12 (forms a complex with Syce2).
(PDF)

S1 File Master data table with graph values. (XLSX)
