# Supplementary figures and images for: Distinct cellular and reproductive consequences of meiotic chromosome synapsis defects in syce2 and sycp1 mutant zebrafish
Source: PLoS Genet. 2025 Sep 5;21(9):e1011656. doi: 10.1371/journal.pgen.1011656 (PMC12425308; doi:10.1371/journal.pgen.1011656)

A.

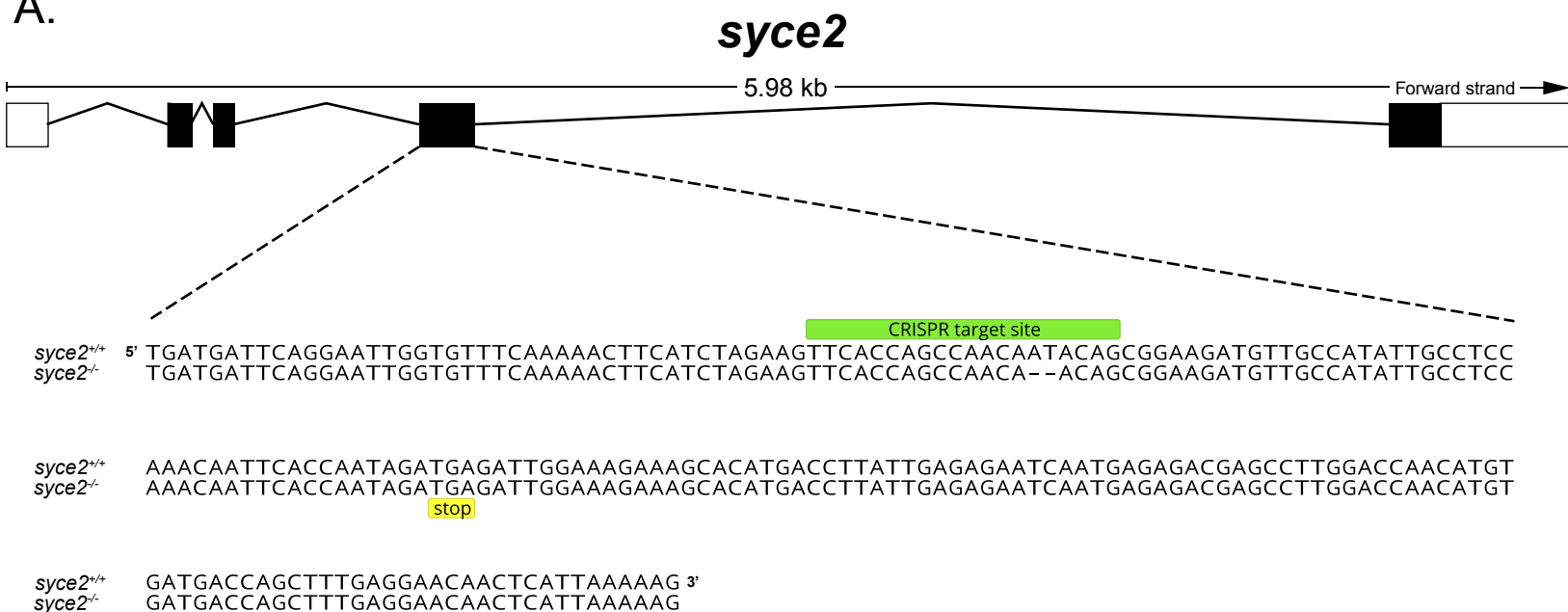

B.

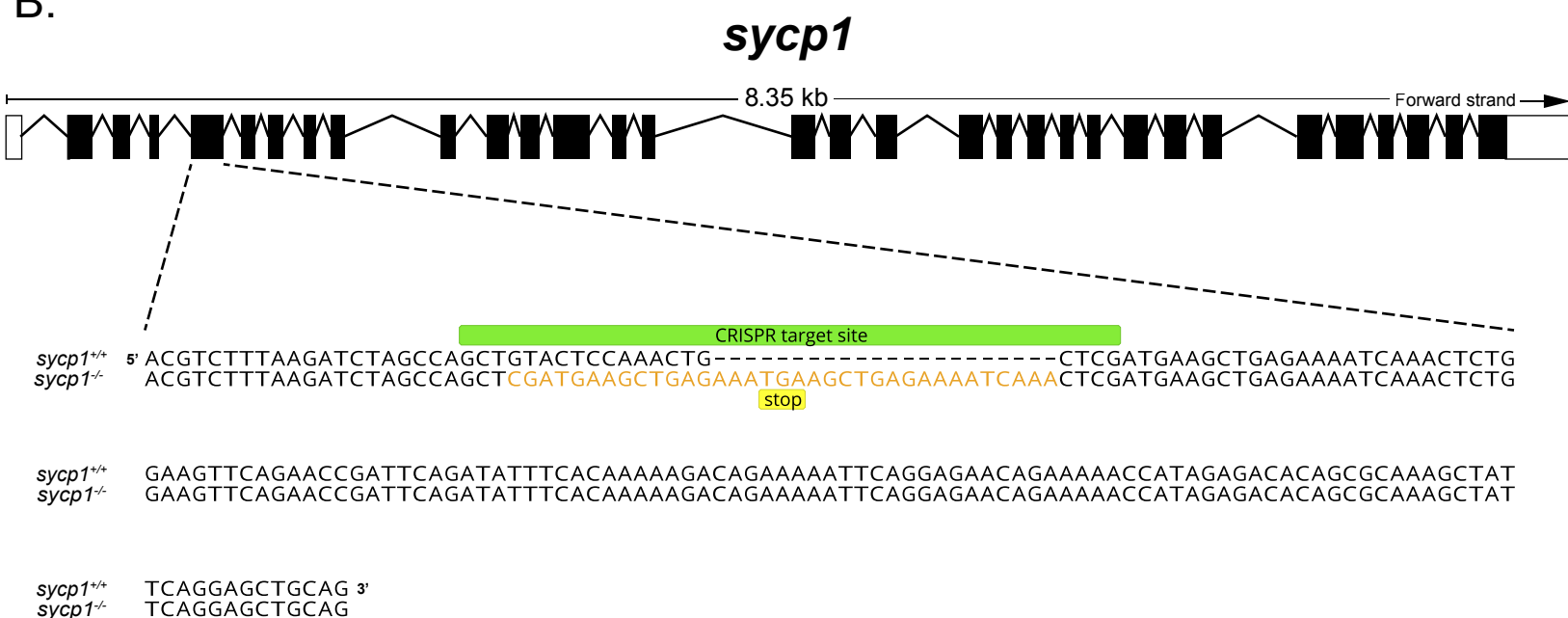

C.

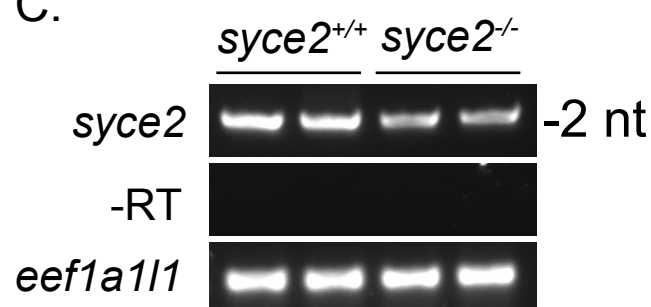

D.

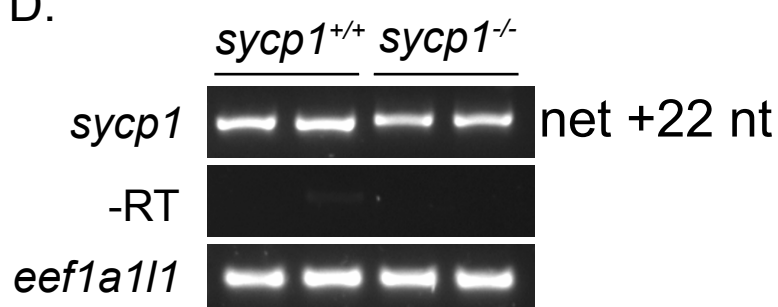

Supplement: S1 Fig — (A) Creation of the syce2 mutation by CRISPR/Cas9 showing a 2 nucleotide (nt) deletion within exon 4 (out of 5). The CRISPR target site (green) and premature stop codon (yellow) are shown. (B) Creation of the sycp1 mutation by CRISPR-Cas9 showing the site of the mutation within exon 5 (out of 32). The CRISPR target site (green), premature stop codon (yellow) and the complex mutation (orange) are shown. The complex mutation leads to a net increase of 22 nt. (C–D) RT-PCR from wild-type, syce2-/- and sycp1-/- testes. Two biological samples were used for each genotype. -RT = no reverse transcriptase. eef1a1l1 is the positive control. (D) Note the slower migration in the sycp1-/- samples due to the complex mutation with a net increase of 22 nt. (PDF) [file pgen.1011656.s001.pdf]

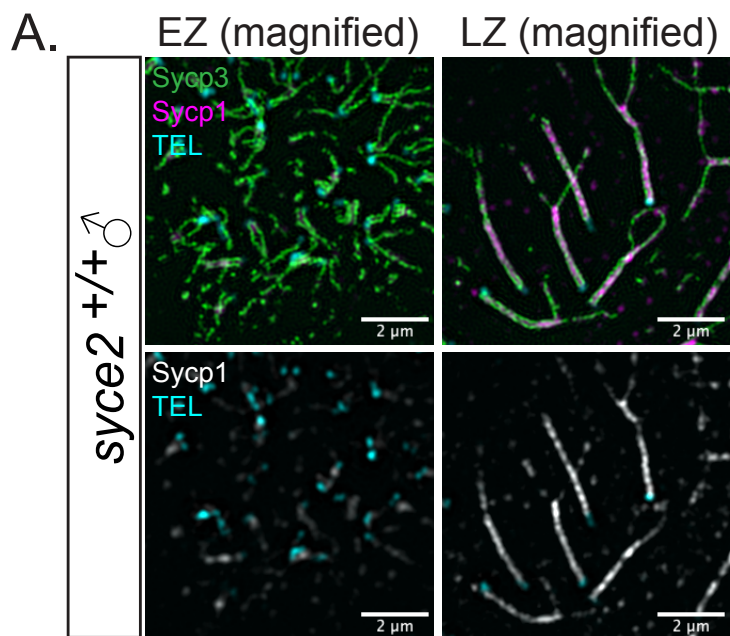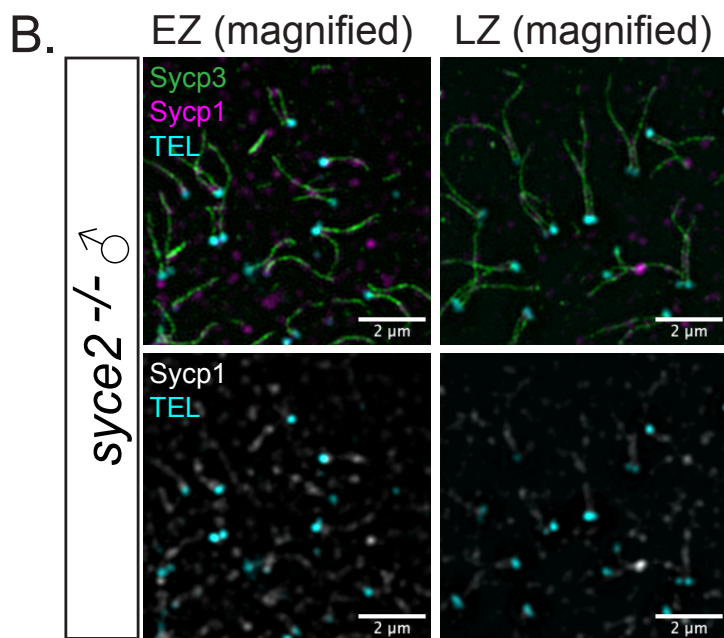

Supplement: S2 Fig — Magnified images from EZ and LZ stages from Fig 2A and 2B. (PDF) [file pgen.1011656.s002.pdf]

*sycp1* <sup>+/+</sup> ♂

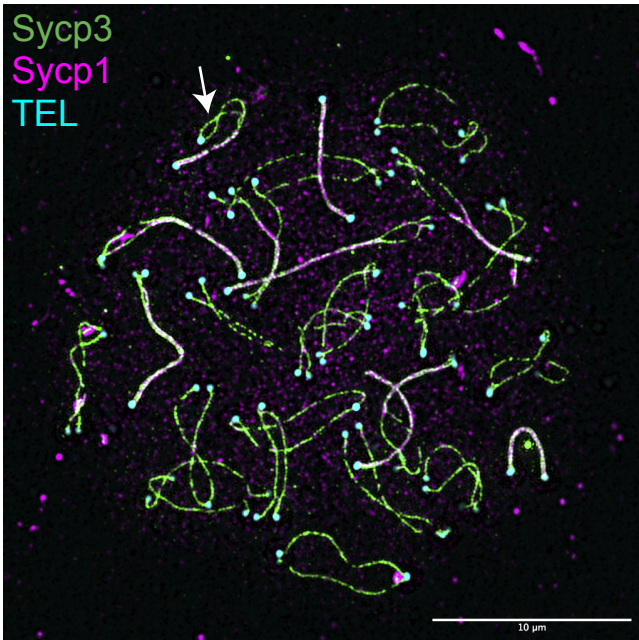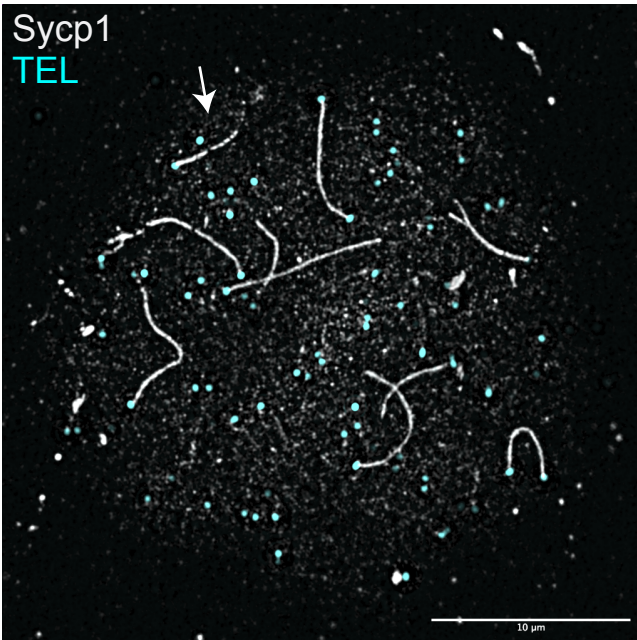

*sycp1* <sup>+/+</sup> ♂

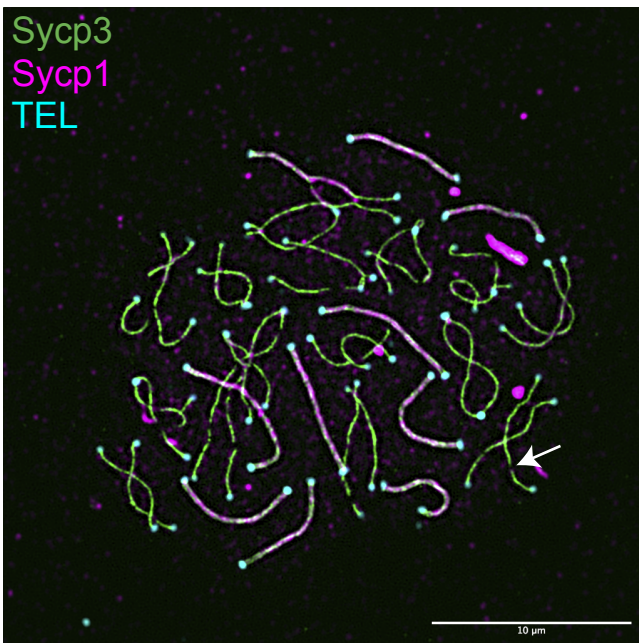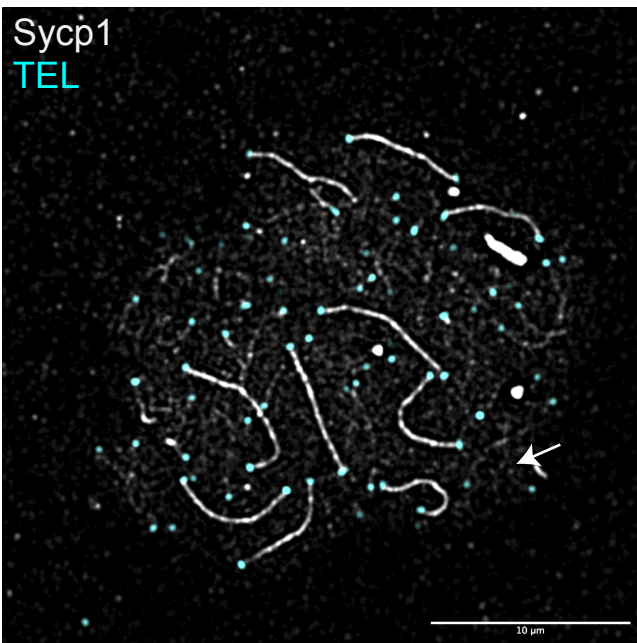

*sycp1* <sup>+/+</sup> ♂

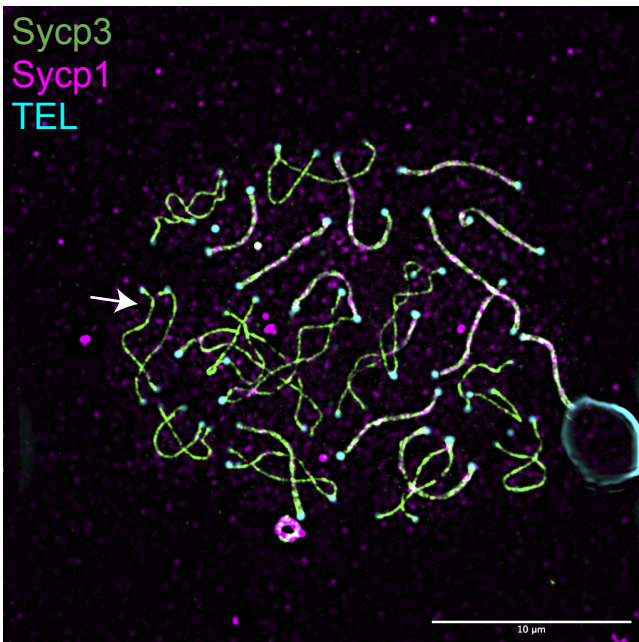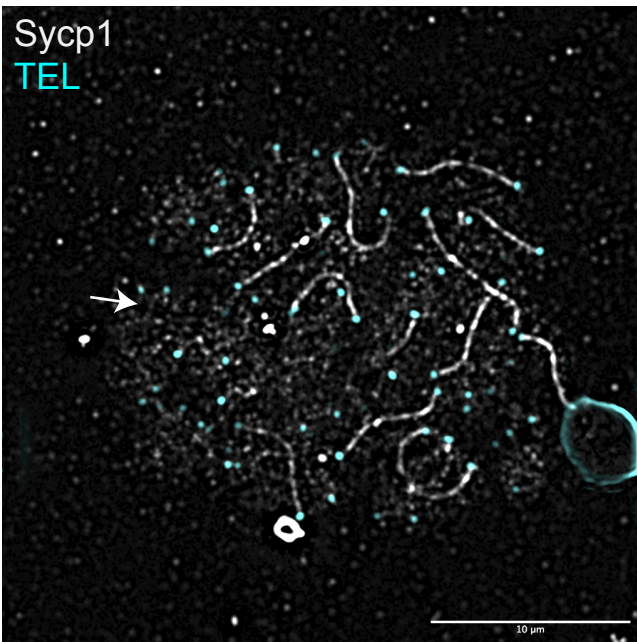

Supplement: S3 Fig — Examples of diplotene spermatocytes from sycp1+/+ males stained for Sycp3 (green), Sycp1 (magenta and gray) and Telomeres (cyan). Spermatocytes were classified as diplotene if they contained three or more bivalents with 50% or more desynapsis. Arrows point to examples of desynapsed sub-telomeric regions that were analyzed for co-alignment (< 0.5 µm). Scale bar = 10 µm. (PDF) [file pgen.1011656.s003.pdf]

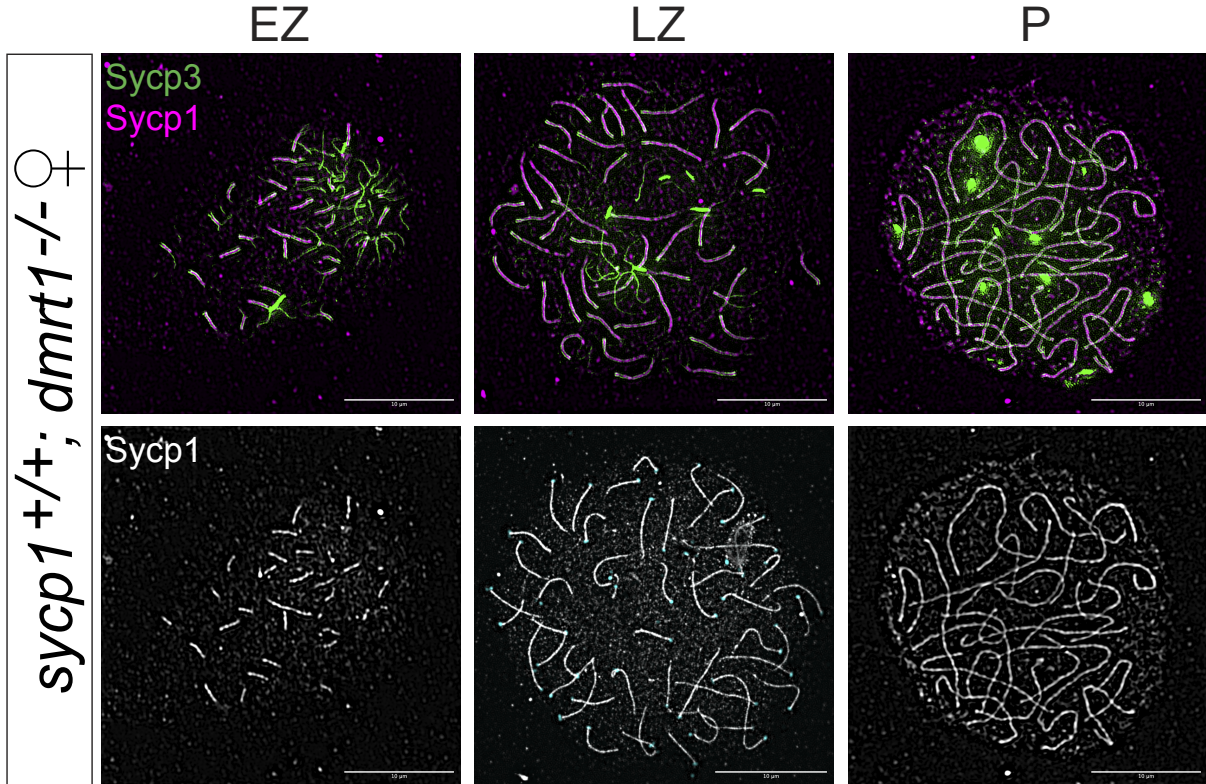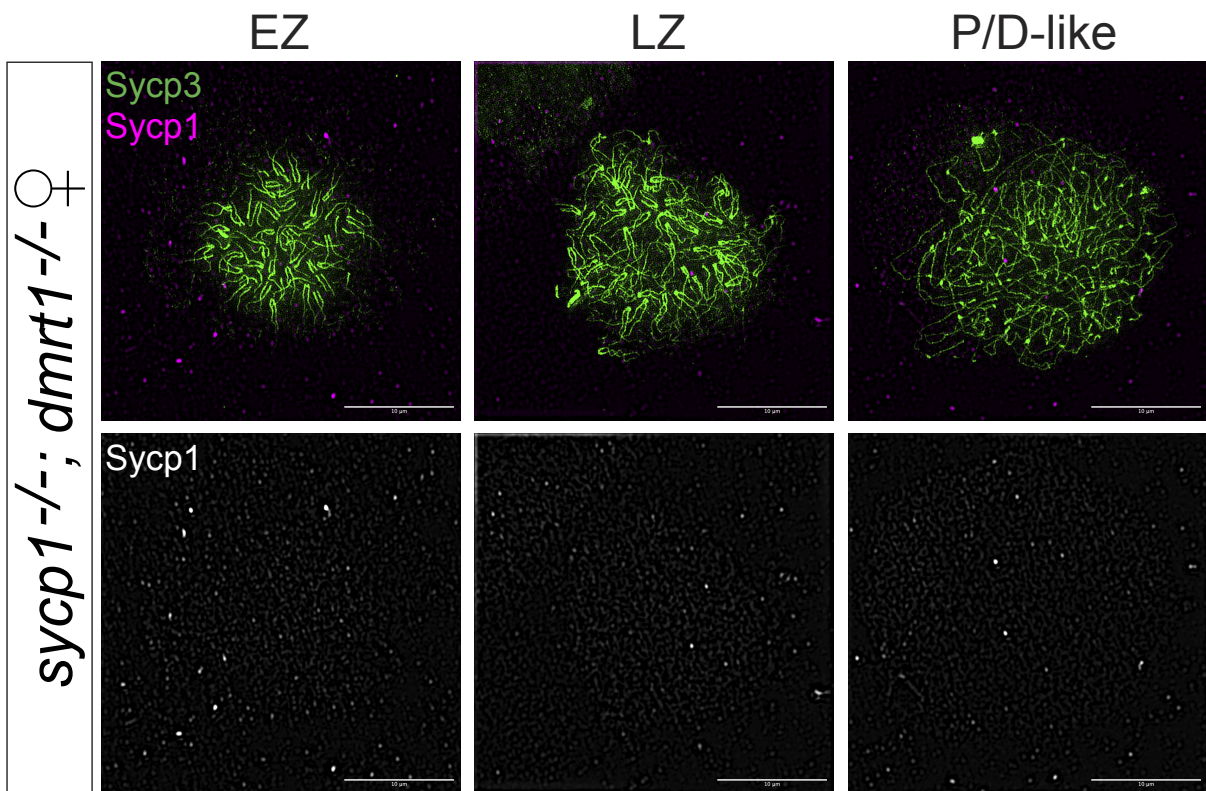

Supplement: S4 Fig — (A–B) Surface-spread chromosomes from sycp1+/+; dmrt1-/- (A) and sycp1-/-; dmrt1-/- (B) oocytes stained for Sycp3 (green) and Sycp1 (magenta and gray). Examples of spread chromosomes from meiotic prophase described in Fig 3 A–B. Scale bar = 10 µm. (PDF) [file pgen.1011656.s004.pdf]

A. *syce2* WT      *syce2* MUT

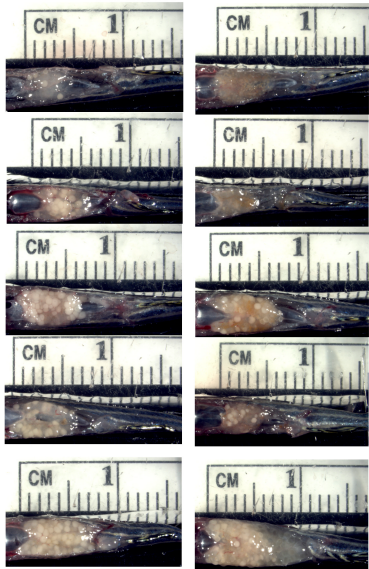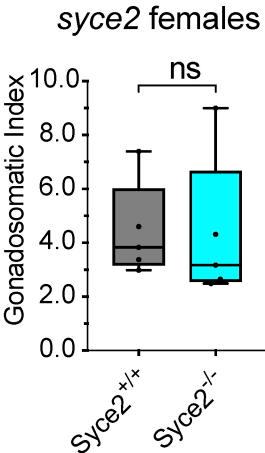

B. *syce2* WT      *syce2* MUT

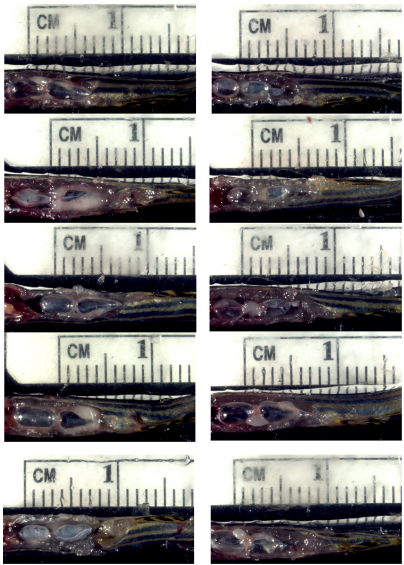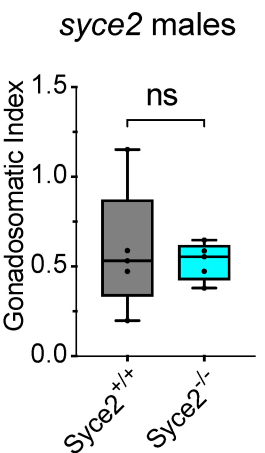

Supplement: S5 Fig — Brightfield images of ovaries (A) and testes (B) in situ that were used to calculate the gonadosomatic index (GSI) for each sex. (PDF) [file pgen.1011656.s005.pdf]

A. *sycp1; dmrt1*  
+/+; -/-      -/-; -/-

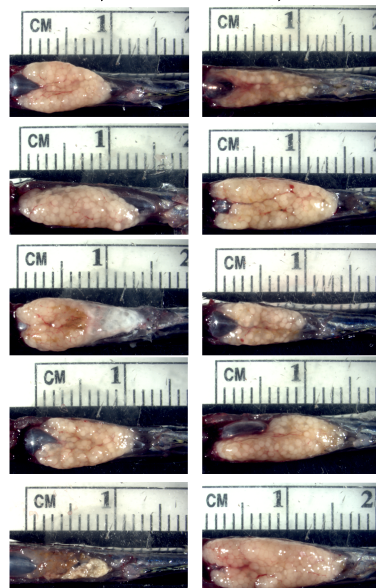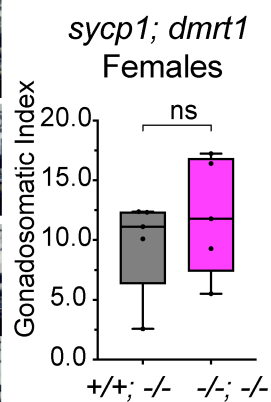

B. *sycp1* WT      *sycp1* MUT

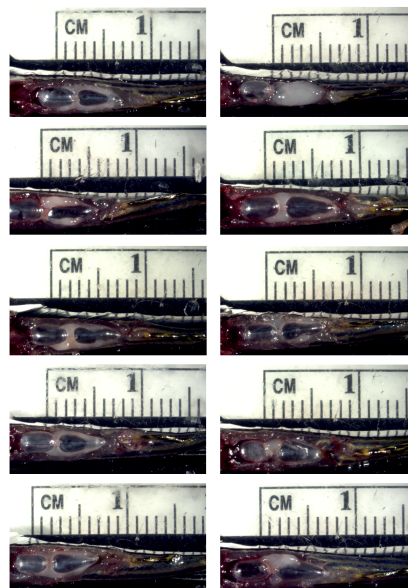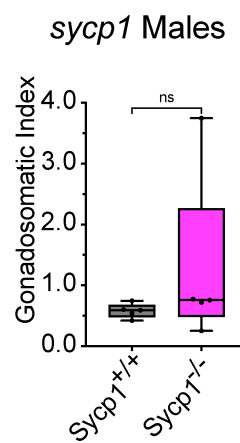

Supplement: S6 Fig — Brightfield images of ovaries (A) and testes (B) in situ that were used to calculate the gonadosomatic index (GSI) for each sex. Graphs are the same as in Fig S5. (PDF) [file pgen.1011656.s006.pdf]

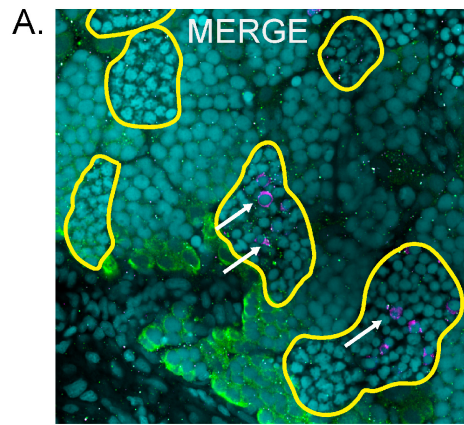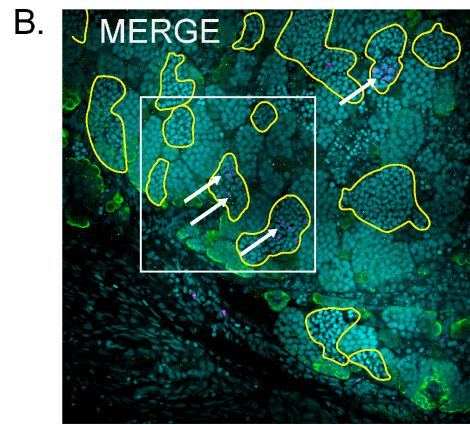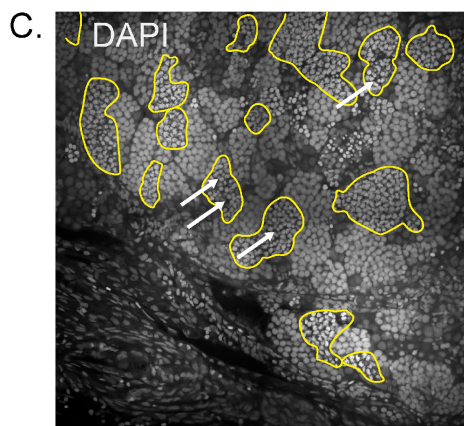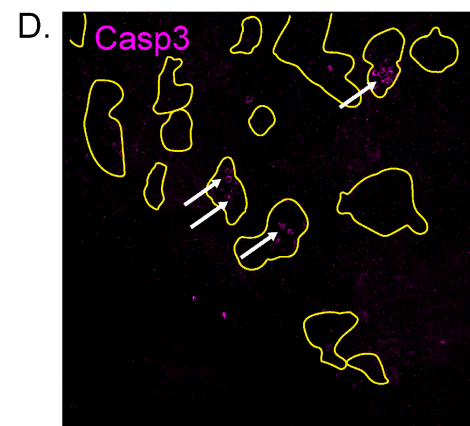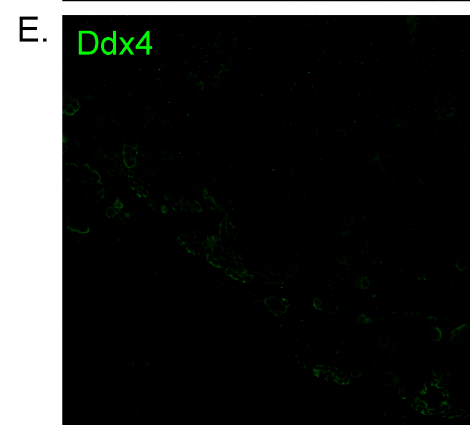

Supplement: S7 Fig — Four stitched images showing the germ cell landscape of a sycp1-/- testis with examples of cleaved Caspase-3 stained cells (arrows). (A) Testis region stained with DAPI (cyan), cleaved Caspase-3 (magenta), and Ddx4 (green). Patches of cells in metaphase I or metaphase II outlined in yellow. A subset of cells in the outlined regions are positive for cleaved Caspase-3 staining. (B) Larger field image showing the location of the cells in the testis in part A. (C) Same panel as in part B showing DAPI (gray). (D) Same panel as in part B showing cleaved Caspase-3 (magenta). (E) Same panel as part B showing Ddx4 (green). (PDF) [file pgen.1011656.s007.pdf]

A.

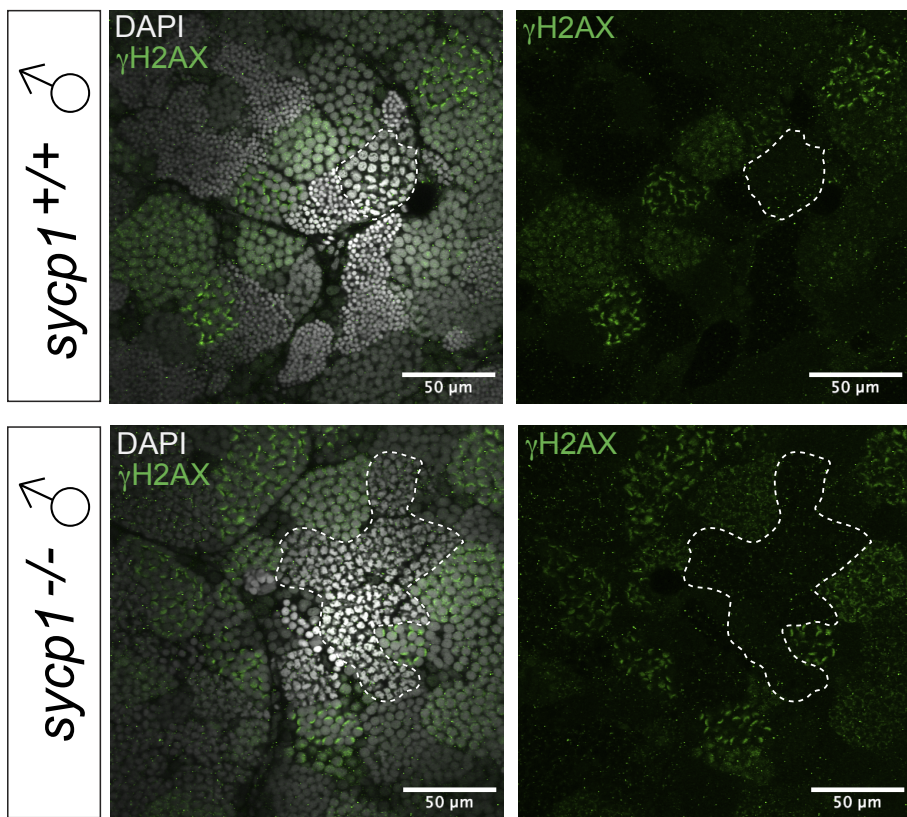

B.

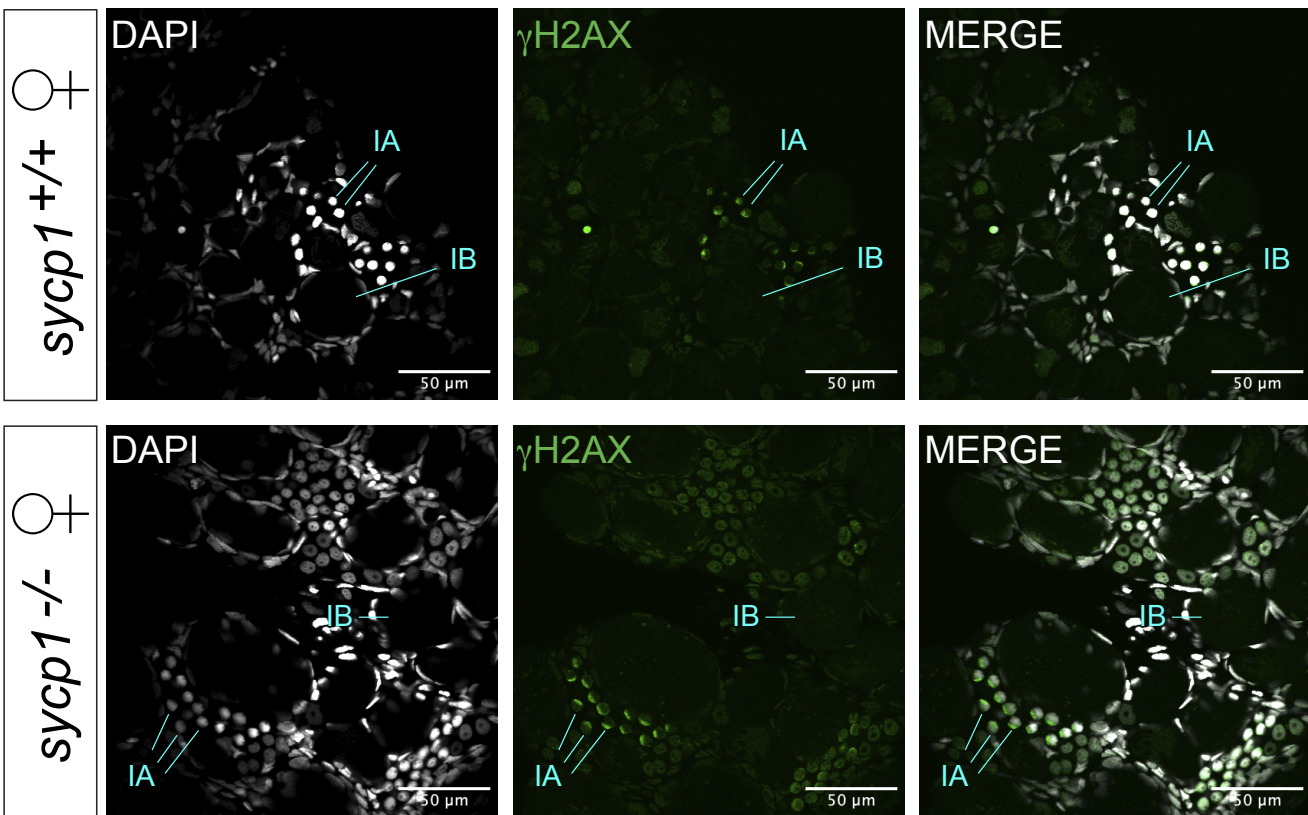

Supplement: S8 Fig — (A) Whole mount testes from adult (> 60 dpf) sycp1+/+ and sycp1-/- males stained with DAPI (gray) and 𝛾H2AX (green). Dashed lines represent metaphase nuclei. Scale bar = 50 µm. (B) Whole mount ovaries from 39 dpf sycp1+/+ and sycp1-/- females stained with DAPI (gray) and 𝛾H2AX (green). Examples of Stage IA oocytes (leptotene/zygotene stage) and Stage IB oocytes are indicated by blue lines. Scale bar = 50 µm. (PDF) [file pgen.1011656.s008.pdf]

# Prophase I

A. WT

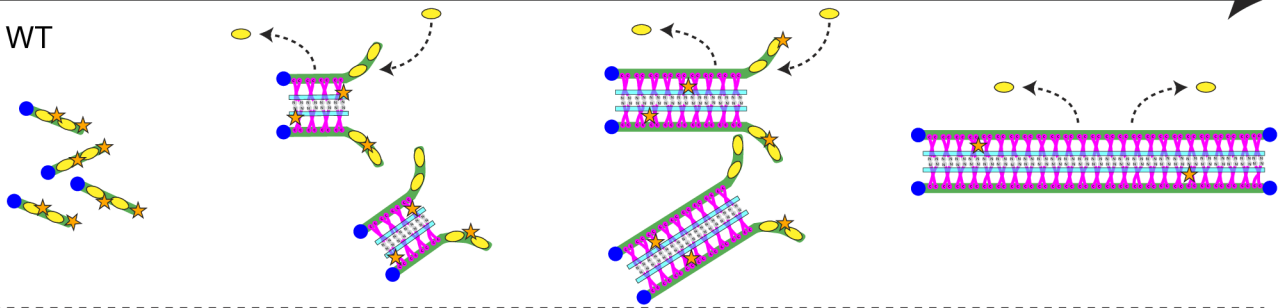

B. *syce2*<sup>-/-</sup>

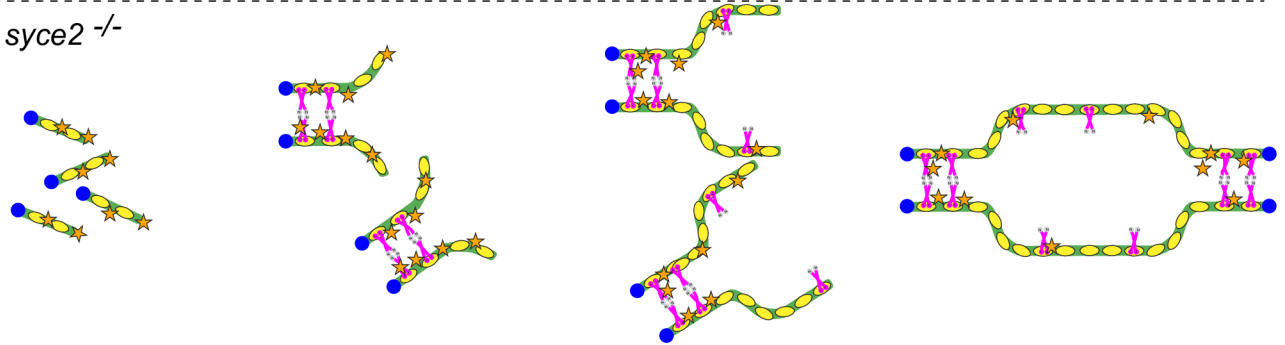

C. *sycp1*<sup>-/-</sup>

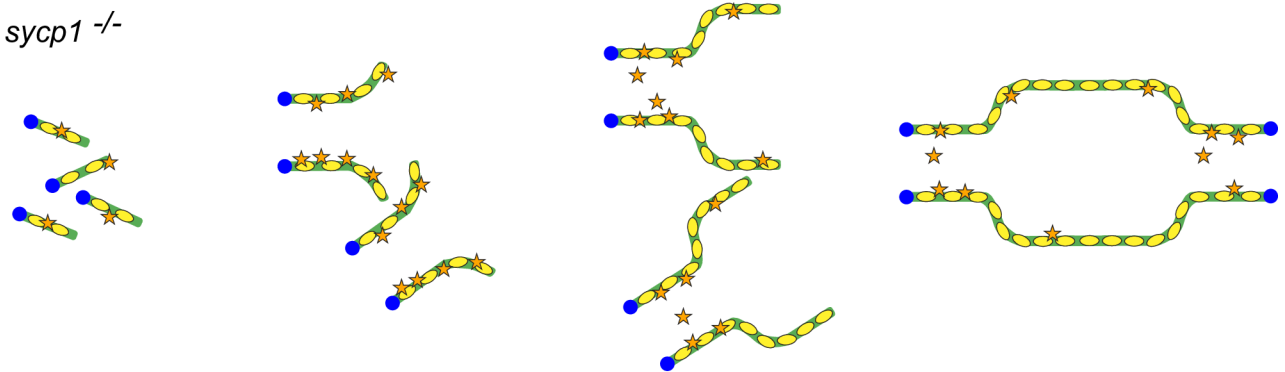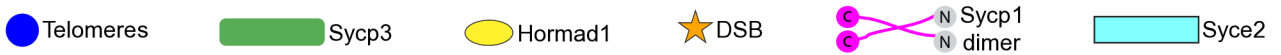

Supplement: S9 Fig — (A–C) Temporal progression of chromosome axis formation, DSB repair, Hormad1 localization, synapsis and homolog juxtaposition. (A) In wild-type zebrafish, DSBs form in early meiotic prophase I and are gradually repaired. Hormad1 localizes to asynapsed regions and is displaced upon synapsis. As synapsis progresses, homologs are “zippered up”. (B) In syce2-/- mutants, there is partial recruitment of Sycp1 to synapsed-like regions and unaligned axes. Hormad1 localizes along the entire length of chromosomes. DSBs form but repair is less efficient. Without a fully formed SC, chromosome co-alignment is limited to sub-telomeric regions. (C) In sycp1-/- mutants where there is no synapsis, Hormad1 localizes along entire axes and DSB repair is less efficient. Chromosome co-alignment is also limited to sub-telomeric regions. Not shown are other central element components: Syce1, Syce3, Six6os1, and Tex12 (forms a complex with Syce2). (PDF) [file pgen.1011656.s009.pdf]
